# Rapid Arbitrary‐Shape Microscopy of Unsectioned Tissues for Precise Intraoperative Tumor Margin Assessment

**DOI:** 10.1002/advs.202511919

**Published:** 2025-11-20

**Authors:** Zhicheng Shao, Xiangwei Meng, Peifen Fu, Keer Huang, Xibei Chen, Yue Ying, Jun Li, Yuxin Zheng, Xiaolong Li, Hyeon Jeong Lee, Jingyi Feng, Yuexin Lu, Runping Cai, Xiongling Jiang, Qing Chen, Hong Zhang, Xiaoyong Man, Li Lin

**Affiliations:** ^1^ College of Biomedical Engineering and Instrument Science Zhejiang University Hangzhou 310027 China; ^2^ The First Affiliated Hospital Zhejiang University School of Medicine Hangzhou 310006 China; ^3^ The Second Affiliated Hospital Zhejiang University School of Medicine Hangzhou 310009 China

**Keywords:** high‐speed pathology, robotic‐driven microscopy, tumor margin examination

## Abstract

Rapid and accurate intraoperative examination of tumor margins is crucial for precise surgical treatment, yet current methods are limited by incomplete tissue sampling and time‐consuming sample sectioning. The Rapid Arbitrary‐Shape Microscope (RAM) is developed, a bedside imaging system that enables high‐speed, 3D microscopy of irregular tissue surfaces without sectioning, providing cellular‐resolution images within minutes while preserving tissue morphology post‐excision. RAM precisely integrates a 3D scanning module with a robotic platform, seamlessly combining morphological measurements with robotic‐driven pathological microscopy. In studies involving metastatic tumors in 39 mouse liver and spleen samples, RAM demonstrated high accuracy in detecting positive margins containing cancer cells, achieving a sensitivity of 99.1% and a specificity of 82.9%. The system's capability is further validated on 12 skin cancer samples from 10 human subjects using nondestructive imaging, highlighting its potential to reduce surgical time and minimize the risk of overlooking residual cancer at surgical margins during intraoperative evaluation.

## Graphical abstract

This study presents a novel microscopic imaging system capable of rapid, section‐free scanning of irregular tissue surfaces, delivering high sensitivity for detecting cancer cell clusters during intraoperative tumor margin assessment.

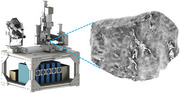

## 1 Introduction

Alongside rapid advancements in clinical oncology, surgery remains a cornerstone of cancer treatment and one of the most effective approaches. Precision in surgery is critical to ensure complete lesion removal while preserving surrounding healthy tissues, which minimizes functional disruptions and promotes faster patient recovery.^[^ ^1^, ^2^, ^3^ ^]^ Surgeons typically rely on intraoperative tumor margin assessments, collaborating closely with pathologists to evaluate surgical margins before closing incisions. While postoperative assessments using formalin‐fixed paraffin‐embedded (FFPE) tissue slices stained with hematoxylin and eosin (H&E) are considered the gold standard, they generally require several days for diagnostic results. The postoperative identification of positive margins often leads to additional surgeries—occurring in ≈15% of cases—imposing a significant burden on both patients and healthcare systems.^[^ ^4^, ^5^, ^6^ ^]^ A rapid, accurate bedside imaging technique capable of detecting cancer cells at tissue surfaces intraoperatively could facilitate immediate re‐excision of malignant tissues, thereby improving surgical outcomes and reducing the need for follow‐up surgeries.

Several Food and Drug Administration‐approved technologies have emerged to meet the demand for intraoperative surgical margin assessment, such as high‐frequency ultrasonography,^[^ ^7^, ^8^ ^]^ micro‐computed tomography (micro‐CT),^[^ ^9^, ^10^ ^]^ and in vivo near‐infrared imaging with contrast agents that accumulate in tumor‐affected tissues.^[^ ^11^, ^12^, ^13^ ^]^ However, due to the lack of cellular‐level pathological details, these imaging modalities have not yet supplanted the long‐standing dominance of frozen section analysis, a technique introduced over a century ago.^[^ ^14^ ^]^ Frozen section analysis involves rapidly freezing tissue samples, thinly sectioning tissues near the surface, and staining them for microscopic examination. While widely used, this method has been criticized for its prolonged processing times, freezing artifacts in adipose tissue, and incomplete sampling of tissue margins.^[^ ^15^, ^16^ ^]^ As a result, surgical procedures can be delayed by 20–30 min or more.^[^ ^17^ ^]^ In breast‐conserving surgery, for example, pathologists typically select only four to six positions for sectioning,^[^ ^18^, ^19^ ^]^ which limits the thoroughness of margin evaluation and contributes to the high rate of secondary surgeries.^[^ ^20^, ^21^, ^22^ ^]^ In response, there is ongoing intensive development of new technologies to address these challenges.

Innovative technologies developed for intraoperative surgical margin assessment can be broadly categorized into optical and non‐optical imaging modalities. Optical approaches generally aim to detect differences in optical properties between tumors and healthy tissues. Specifically, optical microscopy often uses focused light beams near the sample surface to achieve high lateral resolution at the cellular or subcellular scale, which necessitates exceptionally flat sample surfaces. For instance, confocal microscopy and Raman microscopy typically involve imaging sample slices.^[^ ^23^, ^24^, ^25^ ^]^ Although modalities like optical coherence tomography (OCT),^[^ ^26^ ^]^ light‐sheet microscopy,^[^ ^27^ ^]^ ultraviolet‐photoacoustic microscopy,^[^ ^28^, ^29^ ^]^ and microscopy with ultraviolet surface excitation (MUSE) do not always require sectioning,^[^ ^30^, ^31^ ^]^ they still rely on relatively flat surfaces for effective imaging. These constraints often lead to incomplete margin assessment (e.g., limited to areas in contact with a glass slide) and extended sample processing times.^[^ ^30^, ^32^ ^]^

Among these optical approaches, MUSE employs ultraviolet (UV) illumination (< 300 nm) to simultaneously excite multiple fluorescent stains, enabling rapid, slide‐free histological imaging of nuclei and cytoplasm on tissue surfaces.^[^ ^30^ ^]^ As a wide‐field fluorescence microscopy technique, MUSE has been implemented in dermatopathology,^[^ ^33^ ^]^ neuropathology,^[^ ^34^ ^]^ and tumor margin evaluation.^[^ ^35^ ^]^ However, the limited penetration depth of UV excitation inherently confines MUSE to superficial layers. Recent advances, including multi‐directional deep‐UV fiber‐optic illumination,^[^ ^36^ ^]^ speckle‐pattern structured illumination,^[^ ^37^ ^]^ and deep learning‐based virtual staining,^[^ ^38^ ^]^ have enhanced image clarity and depth tolerance. Nonetheless, MUSE remains fundamentally constrained to relatively planar specimens.

Efforts have sought to address the challenges of imaging unflattened tissue surfaces using various optical strategies. For example, Wang et al. developed a UV photoacoustic microscope for label‐free histopathology of sectioned bone tissue.^[^ ^39^ ^]^ Their method corrected surface contour variations of up to ∼0.2 mm by quantifying photoacoustic signal delays along each B‐scan, although its performance on surfaces with larger height variations has not been demonstrated. Similarly, Zhang et al. integrated OCT with a robotic arm to scan kidney tissue; however, the contour deviations remained within the intrinsic depth of field of OCT.^[^ ^40^ ^]^ To the best of our knowledge, no existing microscopy platform is capable of fully automated and comprehensive scanning of irregular tissue surfaces while preserving pathological information.

In contrast, non‐optical imaging modalities typically detect differences in the physical properties between tumors and healthy tissues. For example, micro‐CT and ex vivo MRI can scan entire samples without requiring a flattened surface,^[^ ^9^, ^10^, ^41^ ^]^ but they lack the ability to provide cellular‐level pathological detail. Similarly, handheld approaches like Margin Probe, ClearEdge, and iKnife assess tumor margins by measuring radiofrequency,^[^ ^42^, ^43^, ^44^, ^45^ ^]^ bioimpedance, or chemical properties, respectively. However, their sensitivity in detecting small positive margins is limited due to low spatial resolution. As a result, while non‐optical methods can cover entire samples, they often fall short of the cellular‐level precision achieved by optical microscopy.

In summary, current technologies for intraoperative tumor margin assessment face notable limitations, including incomplete margin scanning due to the reliance on flat sample surfaces in optical microscopy and low resolution or sensitivity in non‐optical approaches. As a result, emerging technologies have yet to demonstrate substantial advantages over frozen section analysis. An ideal imaging modality would overcome these challenges by rapidly scanning entire tissue samples without the need for surface flattening or complex processing, while providing cellular‐level pathological characteristics of tissue margins comparable to H&E staining.

To address these requirements, we developed the Rapid Arbitrary‐Shape Microscope (RAM), a high‐speed imaging platform that eliminates the need for a flattened sample surface. By integrating morphological measurements of intact surgical tissue from a 3D scanner with the pathological imaging contrast of cell nuclei and cytoplasm provided by a MUSE microscope, RAM can generate microscopic images of unsectioned tissue margins in 3D space within minutes. Using a virtual H&E rendering algorithm, RAM converts MUSE images into H&E‐color images and maps them onto the morphological mesh. This capability enables 3D pathological imaging that is interpretable by surgeons, pathologists, and computer‐aided assessment models.

To evaluate the effectiveness of RAM for rapid and accurate examination of tumor margins, we applied the system to 39 mouse liver and spleen tissue samples containing cancer cells, encompassing 68 positive tumor margins. Following RAM scanning, a double‐blind comparison with FFPE H&E analysis demonstrated a sensitivity of 99.1% (95% confidence interval [CI]: 98.6%–99.4%) and a specificity of 82.9% (95% CI: 81.7%–84.0%) for detecting positive tumor margins. In a clinical study involving 12 skin cancer samples resected from 10 human patients, RAM achieved a sensitivity of 90.75% (95% CI: 89.06%–92.11%) and a specificity of 81.58% (95% CI: 79.2%–83.78%). These findings underscore the potential of RAM to substantially improve the efficiency and accuracy of intraoperative margin assessment, reducing both surgical waiting times and the risk of overlooking cancer cell clusters at resection margins.

## 2 Results

### 2.1 Rapid Arbitrary‐Shape Microscope (RAM)

**Figure 1:**
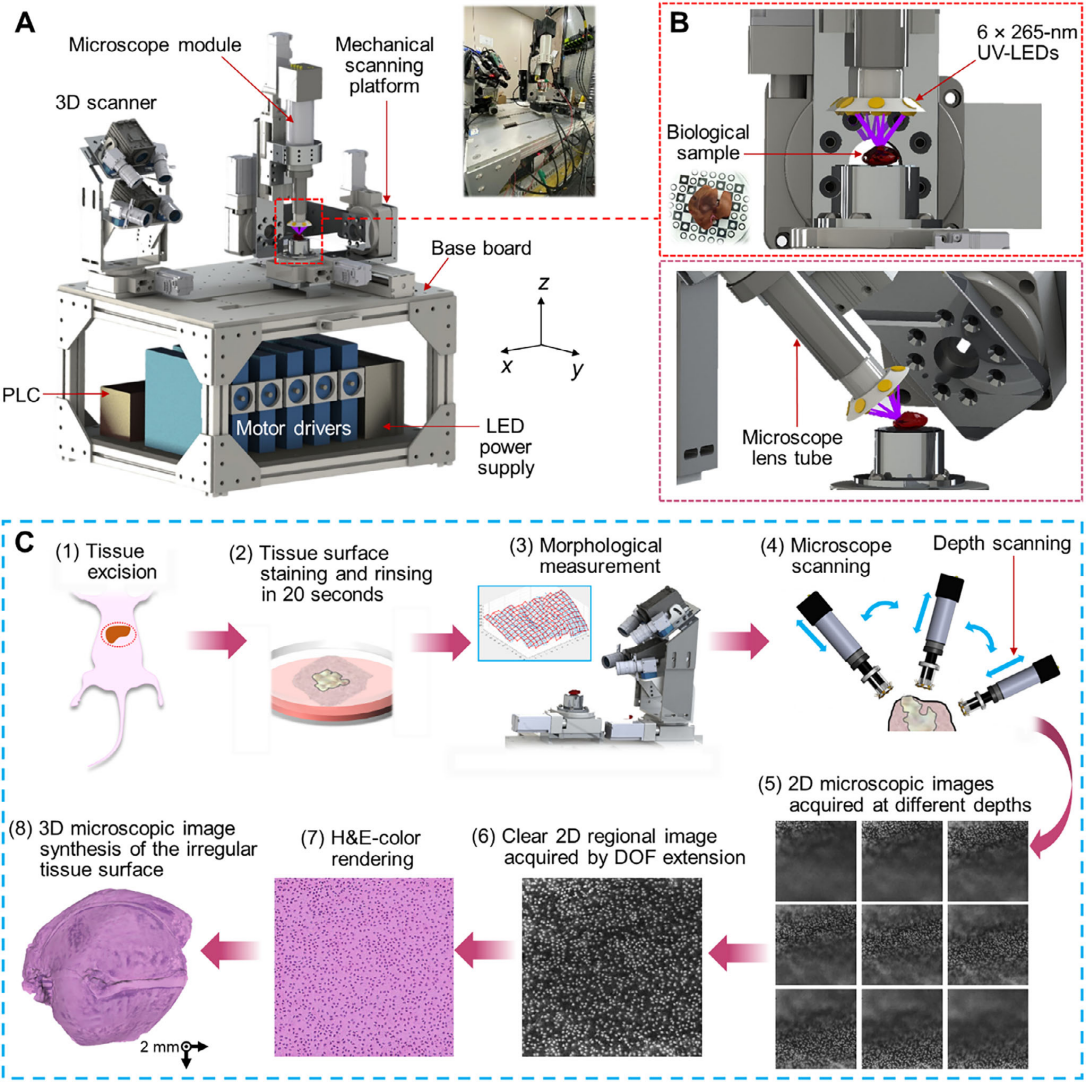
Overview of the RAM system and imaging workflow. A) Schematic and photograph of the RAM system, which integrates a customized 3D scanner, a mechanical scanning platform, a MUSE‐based microscope module, a computer, a programmable logic controller, motor drivers, and an LED power supply. Specifically, the 3D scanner and mechanical scanning platform are mounted on the same base for precise alignment. B) Close‐up view of the UV‐LEDs arranged around the microscope objective, illuminating the biological sample positioned above the calibration chessboard. C) Imaging workflow: (1) Tissue excision from euthanized mice; (2) Surface staining and rinsing of the tissue for 20 s; (3) Morphological measurement using the 3D scanner, dividing the surface mesh into multiple grids and generating mechanical scanning commands; (4) Mechanical scanning of the microscope over the tissue; (5) Acquisition of a cluster of 2D images at varying depths within each surface grid; (6) Image fusion within each cluster using an extended depth of field (DOF) algorithm to produce a clear image; (7) H&E‐like color emulation of the fused image; (8) Cohesive 3D image reconstruction of the irregular surface via an image synthesis algorithm.

The primary objective of this project is to achieve high‐speed imaging of unsectioned tumor margins at the cellular level with high sensitivity. To this end, we developed the RAM system (**Figure** **1**A), which comprises several key modules: a customized 3D scanner, a high‐speed MUSE microscope module, a mechanical scanning platform with six degrees of freedom, and 3D image reconstruction algorithms, including an extended depth of field (DOF) algorithm and an image synthesis algorithm. The RAM system leverages the morphology data of the tumor sample, acquired by the 3D scanner, to compute the scanning trajectory (Experimental Section). The mechanical scanning platform further moves the MUSE microscope module to navigate the sample's irregular surface (Figure 1B). 2D images collected from the same region at different depths form an image cluster, which is processed using the extended DOF algorithm to produce a clear 2D regional image (Experimental Section). These regional images are then integrated through the image synthesis algorithm to create a cohesive 3D representation of the irregular surface (Figure 1C). Pseudo‐color rendering can be applied to RAM images using an unsupervised machine learning approach to emulate H&E colors for easier interpretation (Experimental Section).

To enhance the precision of morphological measurements and reduce mesh holes, we developed a 3D scanner equipped with four cameras positioned at different viewing angles. The MUSE microscope utilizes ultraviolet excitation to highlight superficial cell nuclei and cytoplasm stained with fluorescent dyes (Experimental Section). It consists of a high‐speed camera, a long‐working‐distance objective lens, a tubing lens, and six ultraviolet light‐emitting diodes (UV‐LEDs) mounted around the objective lens. Because glass lenses absorb UV light, no optical filter is needed to block the excitation light. The mechanical platform, which incorporates three rotation stages and three linear stages (Experimental Section), allows the microscope module to tilt up to 83.3 degrees from the vertical axis, facilitating thorough microscopic scanning of irregular surfaces.

We initially developed a proof‐of‐concept prototype utilizing a robotic arm in conjunction with a commercial 3D scanner (Figure S1, Supporting Information). This preliminary setup allowed us to conduct phantom tests on a 3D printed model, validating the workflow and demonstrating the RAM concept. A key challenge was the precise integration of the independent coordinate systems of the 3D scanner and the robotic arm. However, building on the insights gained from the initial prototype, we advanced to a functional prototype with customized modules that enabled comprehensive control over morphological measurements and mechanical scanning trajectories. By moving away from the rigid assembly of independent commercial modules with encrypted raw data, we achieved precise alignment and coordinate unification within the functional prototype, thereby enhancing both performance and reliability.

To compensate for errors introduced during manufacturing and assembly, we initialized the RAM system by calibrating both the microscope and the mechanical scanning platform (Experimental Section). We then tested the RAM functional prototype for spatial alignment accuracy and scanning reliability by imaging a 3D‐printed hemisphere and a seashell decoration using white light illumination (Figure S2, Supporting Information). The microscopic image patterns on the 3D surfaces were complete and continuous, demonstrating effective spatial alignment between the 3D scanner and the mechanical platform, along with high accuracy.

Using a 5× objective lens, the RAM functional prototype can image a sample measuring 15 × 15 × 10 mm^3^ in just 3 min (Movie S1, Supporting Information). This process includes 20 s for tissue surface staining and rinsing, 25 s for morphological measurement with the 3D scanner, and 130 s for microscope mechanical scanning. The rapid scanning capability of RAM is attributed to a high data transmission rate of 81 frames per second and a large camera sensor with 5120 × 5120 pixels, each measuring 4.5 microns. This setup provides large snapshot fields of view (FOV) of 4.60 × 4.60 mm^2^, 2.30 × 2.30 mm^2^, and 1.15 × 1.15 mm^2^ for the 5×, 10×, and 20× objective lenses, respectively. The spatial resolution is determined by the numerical aperture of the objective lens and the sensor's pixel size relative to the magnification. Specifically, the resolution of the 5× objective lens is measured to be 2.0 micrometers (Figure S3, Supporting Information), while the 10× objective refines the resolution to 1.0 micrometer. Surgeons and pathologists can switch to higher magnifications to focus on high‐risk margins identified during the low‐magnification but rapid scan. Additionally, the extended DOF algorithm operates concurrently with the microscopic scanning, providing a 3D microscopic image within 5–10 s after the scan is completed.

### 2.2 Combination of RAM with MUSE

RAM is essentially an imaging platform that combines 3D morphological measurement with biological microscopy. Among various imaging modalities, we chose to pair MUSE with RAM to capitalize on its high imaging speed and cellular contrast. MUSE employs UV light to simultaneously excite conventional fluorescent stains while restricting the excitation depth to the sample surface, aligning with the objective's limited DOF.^[^ ^46^ ^]^ Accordingly, the staining and rinsing procedure primarily focuses on processing the tissue surface. In this study, Hoechst 33342 and Rhodamine B were used for staining cell nuclei and cytoplasm, respectively, both selected for their absorption peaks near 265 nm,^[^ ^30^ ^]^ matching the LED illumination wavelength. In contrast to other published MUSE systems that use color cameras with filtered pixels for red, green, and blue wavelengths,^[^ ^30^, ^31^ ^]^ we opted for a monochrome camera to enhance the signal‐to‐noise ratio (Figure S4, Supporting Information) and reduce the data processing workload, which is particularly beneficial given the limited exposure times required for high‐speed scanning.

**Figure 2:**
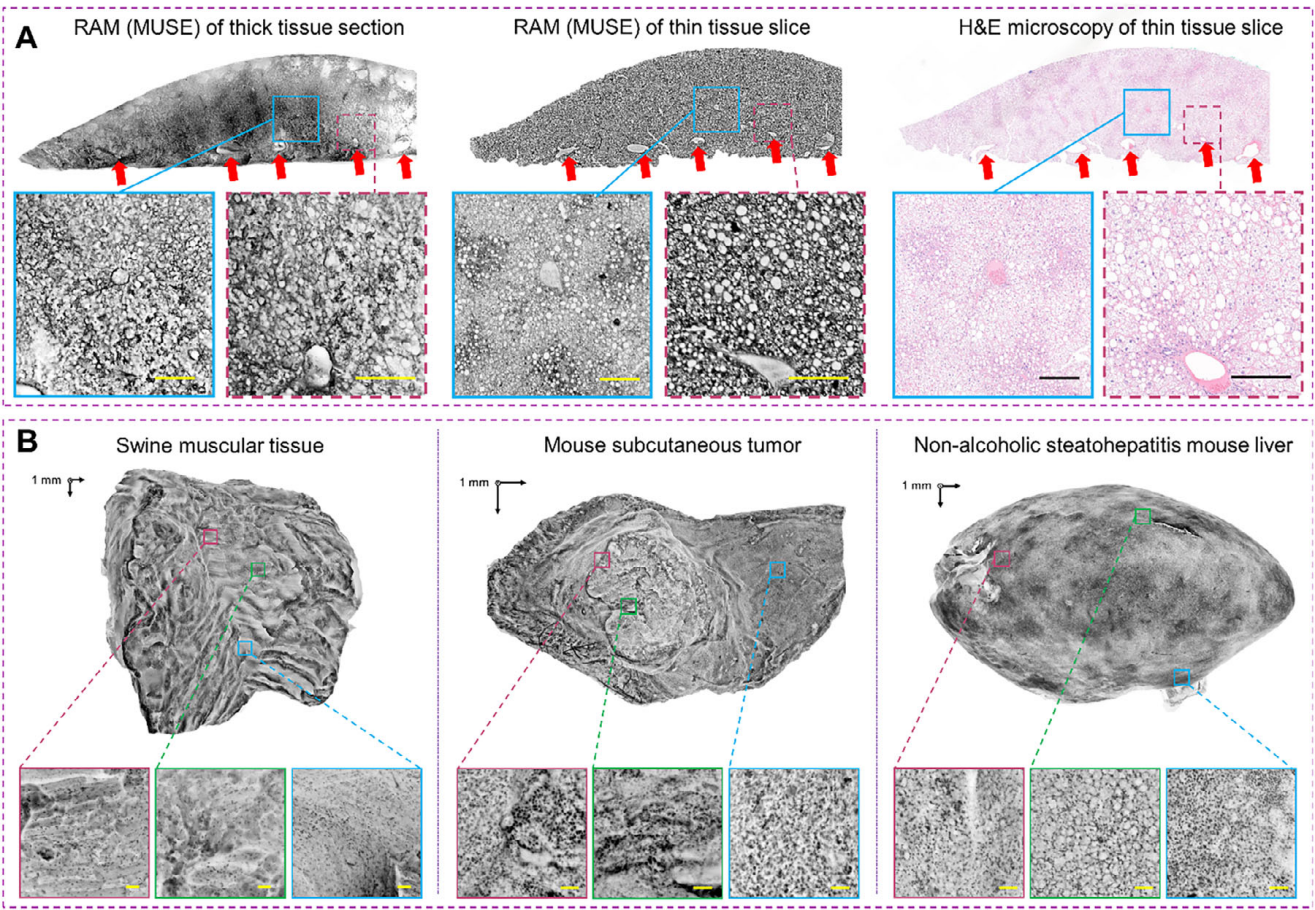
Validation of the RAM system for pathological imaging. A) RAM images of a 1‐mm thick liver section (left) and a subsurface slice ≈0.2 mm beneath the surface (middle). Despite differences in section depth, the cellular structures and blood vessel cross sections remain comparable. An FFPE H&E‐stained image of an adjacent slice (right) closely resembles the RAM image, showing similar blood vessels and cellular structures. B) RAM images of tissue samples with irregular surfaces, including swine muscle, a subcutaneous melanoma tumor, and a liver sample from a non‐alcoholic steatohepatitis mouse. Enlarged views of selected regions (highlighted by squares) show cellular structures such as nuclei and cytoplasm for pathological assessment. Note that the close‐up images may be viewed from slightly different angles compared to the full‐surface image. Scale bars: 0.1 mm.

In previous studies, establishing precise structural correspondence between MUSE and FFPE H&E staining on identical sample surfaces has proven challenging, likely due to inherent sampling differences between sectioning methods.^[30,31,35^^]^ To confirm that MUSE can identify pathological structures comparable to those observed with FFPE H&E staining, we imaged a 1‐mm thick liver section from a NASH mouse using the MUSE microscope with a monochrome camera. To mitigate potential discrepancies caused by sectioning, the sample was further embedded in paraffin, and two adjacent slices were cut ≈0.2 mm beneath the surface previously imaged by MUSE. One slice was dewaxed using xylene and re‐imaged with MUSE, while the other underwent H&E staining for comparison. Both imaging modalities revealed nearly identical pathological features, such as fat‐infiltrated liver cells and blood vessel cross‐sections (**Figure** **2**A). Although MUSE images of thicker tissue segments displayed slightly different background features compared to thin slice images, essential pathological structures remained identifiable and comparable to those seen in H&E‐stained slices. These results demonstrate that MUSE provides sufficient pathological information for effective intraoperative tumor margin assessment.

Utilizing the fully calibrated RAM platform alongside validated MUSE microscopy, we commenced by imaging the surfaces of various biological samples with irregular geometries. These samples included swine muscle tissue, a subcutaneous melanoma tumor, and a liver sample from a non‐alcoholic steatohepatitis (NASH) mouse model. Clear visualizations of muscle cells, melanoma cells, liver cells, adipose cells, and their nuclei were obtained using a 5× objective lens (Figure 2B). Notably, fat infiltration in the NASH liver is distinctly visible in the RAM images. As shown in Movie S2 (Supporting Information), RAM can precisely reveal each cell nucleus on the irregular liver surface using either 5× or 20× objectives. Additionally, the bottom surface of the liver can be scanned by flipping the sample, allowing the 3D scanner to stitch the surface mesh and automatically align the microscopic images across the entire surface. The consistent visualization of cellular features on irregular surfaces further validates the scanning accuracy at a microscopic resolution. To further assess the performance of RAM relative to conventional approaches, we conducted a comparative experiment between RAM and traditional raster‐scanned MUSE (Figure S5, Supporting Information). Imaging results from a piece of swine tissue with an irregular surface demonstrated that RAM achieved superior performance in scanning speed, surface coverage, and 3D image fusion quality compared with raster scanning, which required more scanning steps and suffered from suboptimal viewing angles.

### 2.3 RAM of Intact Tumor Margins

**Figure 3:**
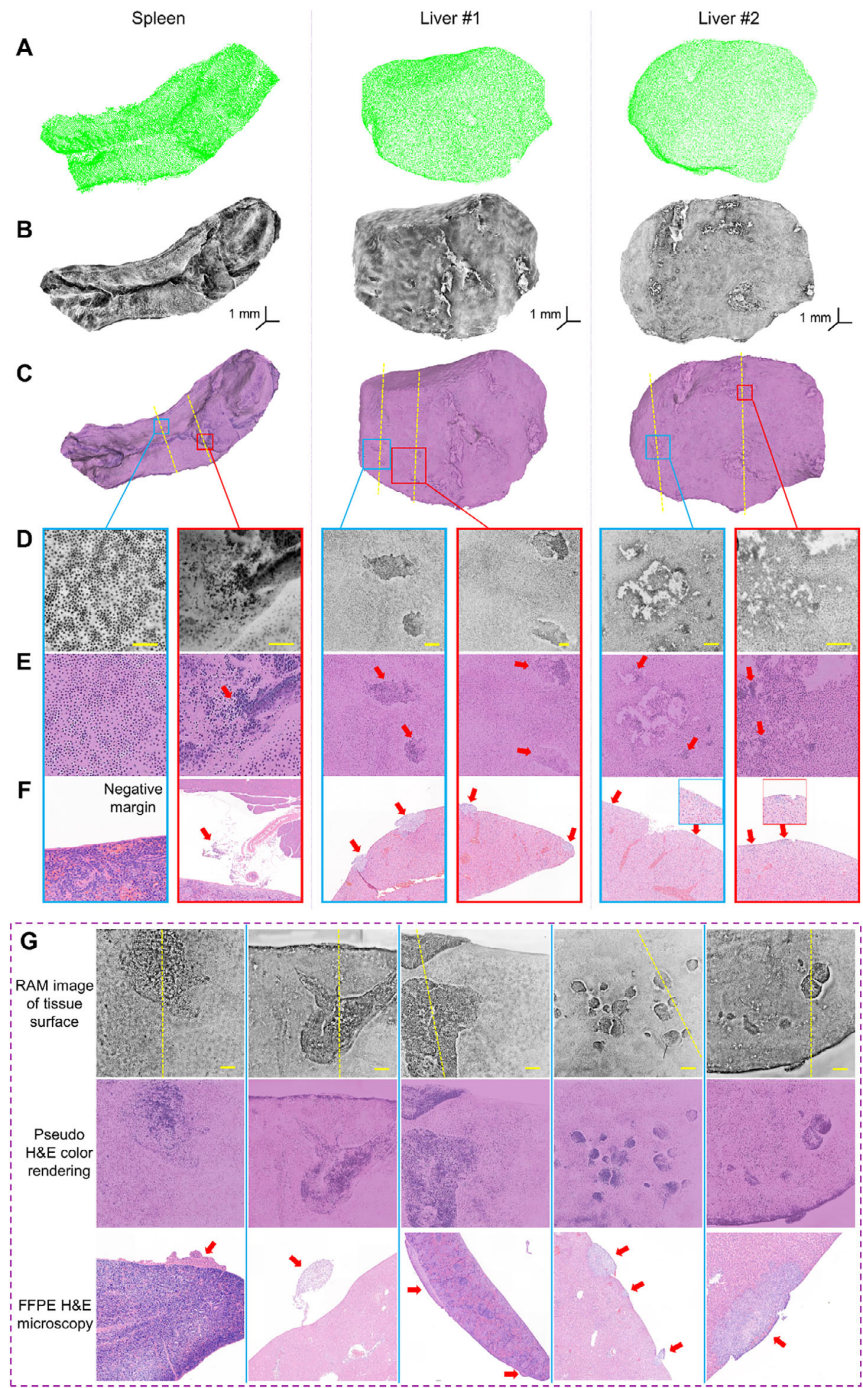
Tumor margin assessment using the RAM. A) Surface mesh of a spleen sample and two liver samples as measured by the 3D scanner in RAM. B) RAM images of the spleen and liver samples, revealing cellular structures on the surface without the need for tissue sectioning. Note that the fluorescence signal intensities in the RAM image pixels are inverted to make cell nuclei appear darker than the cytoplasm. C) H&E‐like rendered images after post‐processing of the raw RAM images. D,E) Close‐up views of regions indicated by the blue and red boxes in (C), displaying pathological features of positive and negative tumor margins. Scale bars: 0.2 mm. F) FFPE H&E‐stained images of tissue slices corresponding to the regions of interest, marked by yellow dashed lines in (C), validating the positive (marked by red arrows) and negative tumor margins identified in the RAM images. G) Additional metastatic tumor margins revealed by RAM, along with the corresponding FFPE H&E‐stained images obtained by vertically sectioning along the yellow dashed lines. Note that some tumor cells near the surface may become dislodged from the tissue after sectioning. Scale bars: 0.5 mm.

The primary goal of developing RAM is to sensitively reveal cancer cells in unsectioned tumor margins with cellular‐level precision. We initially removed subcutaneous masses to create positive tumor margins, yet some of these solid tumor margins were visible to the naked eye (Figure S6, Supporting Information), reducing the necessity for precise intraoperative microscopy. Accordingly, we inoculated either breast tumor cells (4T1) or melanoma cells (B16) into the abdomens of female nude mice, allowing the tumors to grow in the spleens and livers.^[^ ^47^ ^]^ After euthanizing the mice (Experimental Section), RAM was employed to scan a total of 20 mouse spleen and 19 liver samples with irregular surfaces. Using a 5× objective lens for rapid scanning, RAM provided clear visualization of cancer cell clusters without the need for surface flattening or destructive sectioning (**Figure** **3**), enabling precise intraoperative localization of positive tumor margins. Surgeons or pathologists can further switch to a 20× objective lens for a more detailed view of high‐risk regions (Movie S2, Supporting Information).

To evaluate the accuracy of tumor margin detection, we compared RAM findings with FFPE H&E, which is regarded as the gold standard for pathological assessment. Following RAM scanning, all surface areas exhibiting image features indicative of positive tumor margins (Table S1, Supporting Information) were marked and vertically sectioned (along the yellow dashed lines in Figure 3C,G) for comparison with FFPE H&E (Figure S7, Supporting Information). Additionally, random sectioning was performed in areas outside these suspicious regions for negative validation against FFPE H&E. The results demonstrated a high concordance between the margins identified by RAM and those observed in FFPE H&E. To facilitate comparison with H&E staining, we inverted the signal intensities in RAM image pixels to make cell nuclei appear darker than the cytoplasm. Among the positive tumor margins identified by both methods, 4 (5.9%) had a long axis of less than 0.3 mm, and 13 (19.1%) had a long axis of less than 0.6 mm. Notably, RAM was able to detect positive tumor margins that were otherwise not visible to the naked eye (Figure S8, Supporting Information).

From a mesoscopic perspective, healthy liver and spleen tissues exhibit a more homogeneous cellular distribution compared to cancerous tissues. At the cellular level, spleen cell nuclei typically have a fusiform shape, while hepatic cell nuclei are more circular. In contrast, positive tumor margins—characterized by the presence of 4T1 or B16 cells—display a higher nucleocytoplasmic ratio, disorganized nuclear arrangement, and altered cellular structures (Figure 3D–G). The increased nucleocytoplasmic ratio and nuclear aggregation lead to greater dye absorption, resulting in enhanced fluorescent signal intensity at positive tumor margins. Notably, hemorrhagic necrosis and minor scratches from tissue dissection can cause localized fluorescence enhancement similar to that observed at positive tumor margins, but they typically manifest as increased fluorescence outside the cell nuclei. In traditional H&E pathology, distinguishing tumor cells involves identifying a higher nucleocytoplasmic ratio and pathological structure alterations—features that are also evident in the RAM images of positive tumor margins.

### 2.4 Pathological Features Characterized by RAM

Before conducting quantitative and statistical analyses, a visual assessment of tumor margins was performed by four independent surgeons, who evaluated divided RAM images measuring 4.60 × 4.60 mm^2^ for localized assessment. During a brief training session with representative RAM images (two positive and two negative tumor margin examples), the surgeons independently evaluated six RAM image features: nucleocytoplasmic ratio, nuclear density, regional nuclear signal intensity, cellular structural alteration, nuclear morphology heterogeneity, and nuclear arrangement regularity (Table S1, Supporting Information). Following the training, each reader, blinded to any prior information, evaluated and scored these features in the remaining 72 test images. The feature scores from each reader were collected, and histograms for each feature were generated (Figure S9, Supporting Information), revealing significant differences between positive and negative tumor margins.

**Figure 4:**
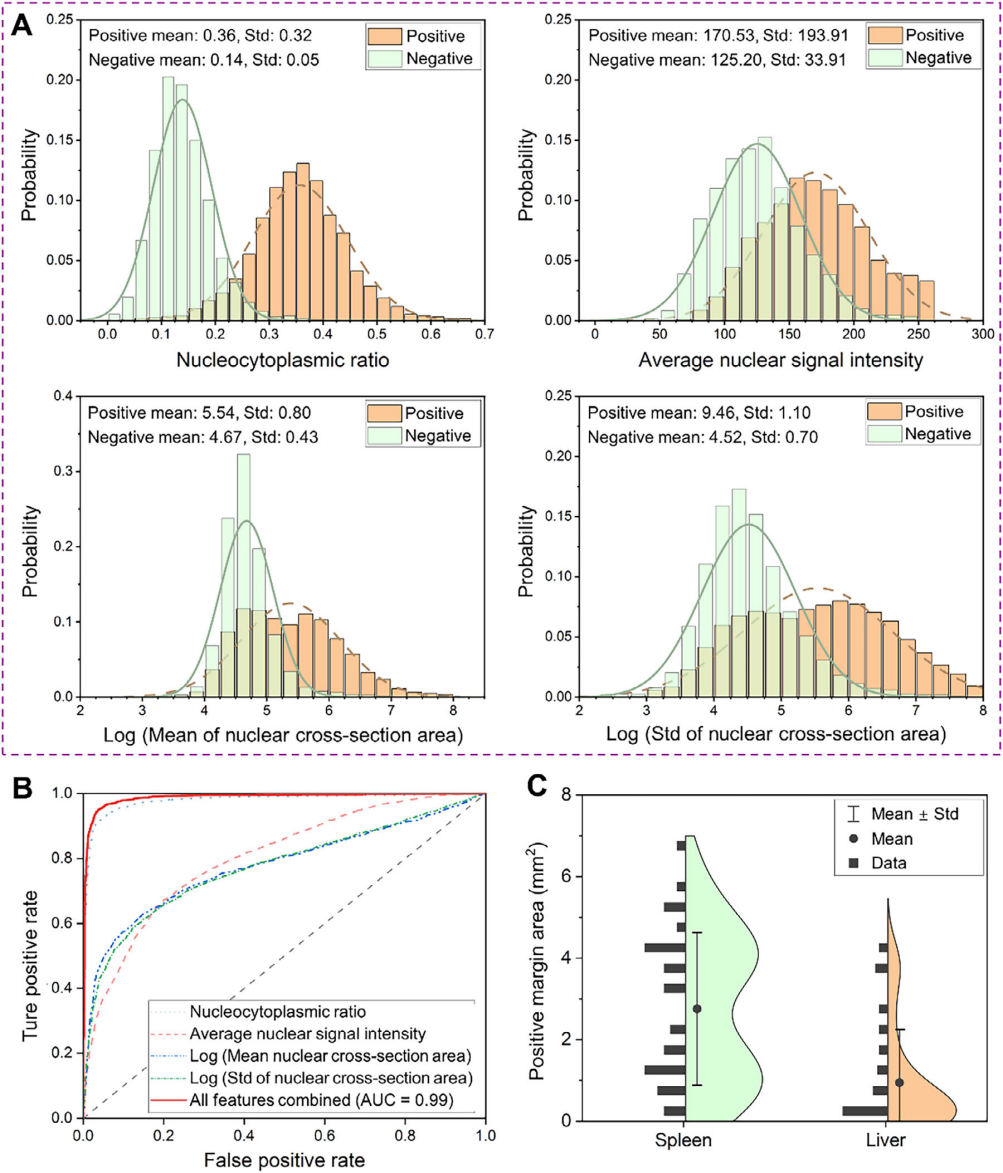
RAM statistics for mouse tissue samples. A) Histograms and Gaussian fitting curves for the four image features in the computer‐aided diagnostic analysis: nucleocytoplasmic ratio, average nuclear signal intensity, mean of nuclear cross‐section area (in pixel numbers), and standard deviation (Std) of nuclear cross‐section area (in pixel numbers). B) Receiver Operating Characteristic curves for tumor margin assessment models based on individual imaging features and a combined model using all features. The areas under the curves for these models are 0.98, 0.80, 0.78, 0.77, and 0.99, respectively. C) Distribution of positive margin areas in liver and spleen samples. The smallest tumor margin detected by RAM measured ≈0.014 mm^2^.

To facilitate rapid assessment during intraoperative examinations, we implemented a computer‐aided diagnostic (CAD) method for the sensitive identification of positive tumor margins. Unlike human visual assessments, which rely on subjective interpretation, the CAD approach quantitatively analyzes images based on distinct image features. For CAD analysis, 3D RAM images were divided into 128 × 128‐pixel subimages, from which four quantifiable features were extracted (Experimental Section, Table S2, Supporting Information): nucleocytoplasmic ratio, average nuclear signal intensity, mean nuclear cross‐section area, and standard deviation of nuclear cross‐section area. Histograms of these image features, particularly the nucleocytoplasmic ratio, indicated differences between positive and negative tumor margins (**Figure** **4**A).

### 2.5 Accuracy of Tumor Margin Assessment

In this study, we employed RAM to assess tumor margins in 57 cancerous tissue samples. Since tumor margins in subcutaneous masses (18 samples) were often visible to the naked eye, our primary analysis focused on metastatic tumors in 19 liver samples and 20 spleen samples, which were populated with either 4T1 cells (69.1%) or B16 cells (30.9%). After RAM scanning, regions with and without abnormal image features were marked and sectioned for comparison with FFPE H&E staining (Figure S7, Supporting Information). Three independent pathologists confirmed 68 positive and 65 negative tumor margins based on the FFPE H&E findings. All RAM images included in the statistical analysis were acquired at 5× magnification to ensure rapid scanning of the entire tissue surface.

To create training and testing datasets for CAD‐based tumor margin assessment, we divided the raw RAM images near the vertical sectioning lines (e.g., yellow dashed lines in Figure 3) into 128 × 128‐pixel subimages (Experimental Section). Of the 10114 subimages in the training group, we randomly selected 4999 subimages from liver tissue and 5115 subimages from spleen tissue. Within this group, 4412 subimages (43.6%) were associated with positive tumor margins, as confirmed by histopathology, while the remaining 5702 subimages (56.4%) were confirmed as negative margins. A tumor margin assessment model employing logistic regression was developed based on the training dataset (Experimental Section). The remaining 6743 subimages were used for blind testing to evaluate the accuracy of tumor margin detection, with 2896 subimages (42.9%) associated with positive histopathology and 3847 (57.1%) confirmed as negative.

Using the testing dataset, we generated Receiver Operating Characteristic (ROC) curves based on the quantified RAM image features (Figure 4B). The assessment model, using four image features, achieved a sensitivity of 99.1% (95% CI: 98.6%–99.4%) and a specificity of 82.9% (95% CI: 81.7%–84.0%), with an area under the curve (AUC) of 0.99 (Figure 4B). Among the individual features, the assessment model based solely on the nucleocytoplasmic ratio provided the highest accuracy, with an AUC of 0.98. This demonstrates the efficacy of RAM for rapid intraoperative tumor margin assessment, utilizing cellular image features and fast whole‐surface scanning to reliably detect cancer cell clusters. In comparison, visual assessments by the four surgeons achieved a sensitivity of 95.2% (95% CI: 88.4%–99.4%) and a specificity of 76.7% (95% CI: 59.1%–88.2%) with an AUC of 0.84. The superior accuracy of the CAD‐based approach is likely due to its larger training dataset and enhanced ability to detect subtle image features that may be less discernible to human readers.

### 2.6 RAM of Human Skin Cancer

**Figure 5:**
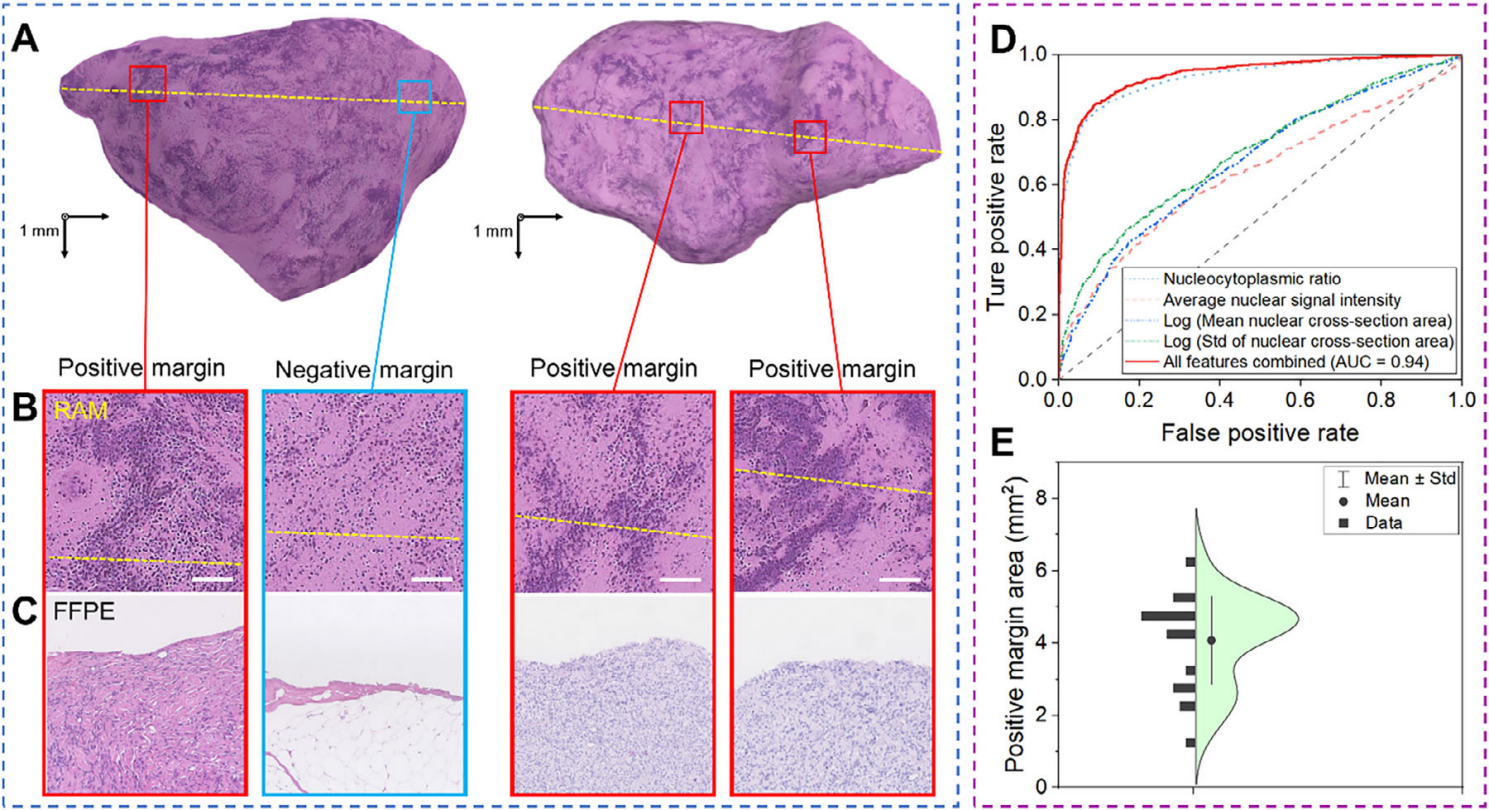
Tumor margin assessment of human skin cancer samples. A) H&E‐like images generated from post‐processed RAM data, providing structural pathological information along the surgical margins. The yellow dashed line indicates the longitudinal axis selected for comparison with the histology section. B) Enlarged views of the regions outlined by the red and blue boxes in (A), highlighting pathological features associated with positive and negative tumor margins, respectively. Scale bars: 0.2 mm. C) Corresponding FFPE H&E‐stained histological sections from the same regions shown in (B), serving as validation of the RAM‐based margin assessment. D) ROC curves for tumor margin classification based on individual image‐derived features, as well as a combined model incorporating all features. E) Distribution of positive margin areas in human skin samples.

Building on our experience with cancerous tissue samples from small animal models, we further evaluated the feasibility of using RAM for detecting positive tumor margins in human skin cancer. Ten patients were recruited from the Second Affiliated Hospital of Zhejiang University, and 12 freshly resected tissue samples were scanned using RAM within minutes, without requiring tissue deformation or sectioning. Because the assessment primarily targeted the surgical surface, samples did not require flipping during imaging. High‐speed microscopic scanning was performed with a 5× objective, providing clear visualization of cell nuclei and cytoplasm along the resection margins (**Figure** **5**). The tissue samples were subsequently returned for routine histopathological examination, which involved sectioning along the longitudinal axis. This axis was independently annotated on the RAM images in a double‐blind manner, and RAM‐based diagnoses along this line were compared with those obtained from FFPE H&E sections (Experimental Section). A high degree of concordance was observed in cellular structures and tissue types near the longitudinal axis. Key imaging and sample parameters, including RAM scanning time, specimen dimensions, and the ratio of cancer‐positive to cancer‐negative margin areas, are summarized in Table S3 (Supporting Information).

To classify tissue types along the surgical margins of human skin samples, we trained and tested a diagnostic model using the same approach as in the small animal study. RAM images near the sectioning regions were divided into 6239 subimages for model development and evaluation. A logistic regression model, using the same image features as in the small animal experiments, was trained on 2163 subimages labeled as positive tumor margins and 1580 labeled as negative, with ground truth determined by FFPE H&E staining. The remaining 2496 subimages—1398 positive and 1098 negative—formed the test dataset. The model achieved a sensitivity of 90.75% (95% CI: 89.06%–92.11%), a specificity of 81.58% (95% CI: 79.2%–83.78%), and an AUC of 0.94 (Figure 5D). The smallest tumor margin detected by RAM measured ≈1.48 mm^2^, which was larger than that observed in mouse studies due to intrinsic differences in sample characteristics. Mouse tumor margin samples contained scattered metastatic lesions, including cancer cell clusters on the tissue surface. In contrast, the skin cancer samples from human subjects were excised from subcutaneous tumors, typically presenting positive margins with larger affected areas. These results demonstrate high diagnostic accuracy in both preclinical and clinical settings, highlighting the potential of RAM to reduce the risk of overlooking small but positive tumor margins during surgery.

## 3 Discussion

While several slide‐free microscopic imaging modalities have been developed for intraoperative histology, they often require a flat imaging surface, limiting the completeness of tumor margin assessment. To overcome this limitation, we developed RAM, an advanced automated imaging platform that uniquely enables rapid microscopic scanning of irregular surfaces in excised biological samples without tissue deformation or sectioning. The core innovation of RAM lies in its integration of 3D morphology measurement with cellular imaging contrast provided by microscopic modalities like MUSE. To facilitate both rapid and precise scanning, we designed a mechanical scanning platform with six degrees of freedom, allowing for perpendicular scanning of tissue surfaces regardless of their orientation. RAM operates by dividing the surface mesh from the 3D scanner into grids corresponding to the microscope's FOV, planning scanning trajectories, and capturing images from each grid. An extended DOF algorithm processes images acquired at various depths, producing clear 2D images for each grid. These images are then synthesized to create a comprehensive 3D surface image. All key modules of the functional prototype were custom‐developed, enabling seamless coordination of control signals, data transmission, and image processing within a unified framework. Notably, one of the primary technical challenges—precise unification and calibration of the 3D scanner and mechanical scanning platform—was successfully addressed.

Using the calibrated RAM, we scanned a variety of biological samples, including liver, spleen, subcutaneous tumors, muscle, and adipose tissue. RAM provides clear pathological images using 5×, 10×, and 20× objective lenses, offering detailed visualization of cell nuclei and cytoplasm. Typically, the 5× objective is employed for rapid scanning of tissue surfaces, while higher magnifications allow for close examination of high‐risk regions identified during initial scans. To evaluate tumor margin assessment accuracy, we quantified multiple imaging characteristics from RAM images and developed assessment models to distinguish positive tumor margins from healthy tissue. Compared to standard FFPE H&E staining, the computer‐aided approach achieved a sensitivity of 99.1% and a specificity of 82.9%, demonstrating RAM's efficacy as a fast and accurate tool for intraoperative tumor margin assessment without freezing or sectioning. Although our statistical analysis focused on scattered tumor margins in liver and spleen tissues, RAM's performance in imaging subcutaneous tumor margins (Figure S10, Supporting Information) highlights its potential in intraoperative margin assessment during Mohs surgery.

RAM is essentially an open and extensible platform that combines morphological measurement, precise mechanical scanning, optical microscopy, and image synthesis algorithms. Its modular architecture allows the MUSE component to be readily replaced with other imaging modalities—such as OCT, Raman microscopy, high‐frequency ultrasound, or hyperspectral imaging—without substantial changes to the hardware control or image fusion pipelines. This versatility enables RAM to be adapted for a broad spectrum of applications. In biomedicine, it could support rapid pathological assessment during surgery or biopsy, enable large‐area volumetric microscopy, or facilitate multimodal organ imaging. Beyond the biomedical domain, RAM could also be applied in industrial settings, where it may provide high‐precision defect detection in irregular components or deliver detailed surface characterization of delicately engineered products.

Although we progressed RAM from a proof‐of‐concept to a functional prototype with enhanced performance and reliability, several refinements could further accelerate its clinical translation: 1) employing optical approaches to extend DOF and reduce mechanical scanning duration,^[^ ^48^, ^49^ ^]^ 2) automating objective lens switching to save time, 3) minimizing the system's footprint to facilitate its use in operating rooms for human tissue imaging. Additional limitations stem from the shallow imaging depth of MUSE, which is inherently constrained by the limited penetration of ultraviolet excitation. This could be mitigated by incorporating complementary modalities such as OCT or high‐frequency ultrasound. Moreover, because the robotic platform currently scans the microscope only from above, complete surface imaging requires manual inversion of the specimen and subsequent image registration of the top and bottom surfaces. Automating this step with a sample‐flipping module would streamline the workflow and further accelerate acquisition. Despite these potential enhancements, RAM stands as the first imaging modality capable of providing rapid, cellular‐level imaging of irregular surfaces in intact tissue samples. From an application perspective, current clinical validation has been conducted in the dermatology department in accordance with IRB regulations. Moving forward, we aim to extend the study to additional cancer types to evaluate the system's performance across a broader range of clinical scenarios.

## 4 Experimental Section

### System Construction

The RAM system comprises several key components: a 3D scanner, a MUSE microscope, a mechanical scanning platform, and a sample holding plate with a calibration chessboard. Beneath the imaging platform, multiple supplementary modules, including motor drivers, a programmable logic controller, power supplies, and heat dissipation fans, were integrated for effective operation. The system was supported by a computer equipped with a high‐speed data acquisition board (Coaxlink Quad G3, Euresys Inc.) and an image processor (GEFORCE RTX 4080, NVIDIA Inc.), enabling fast data streaming and processing.

(1) 3D scanner: In the proof‐of‐concept prototype, a commercialized 2‐camera 3D scanner was employed to measure the morphology of biological samples with wet surfaces. However, this model lacked the precision needed to generate hole‐free meshes, especially given the high‐speed and accuracy requirements for tumor margin scanning. These issues were addressed in the functional prototype by developing a 3D scanner that incorporates four cameras (MV‐CS020‐10UM, Hikrobot Inc.) with higher numerical aperture lenses. Two projectors (TX‐X20, Tengju Co.) were integrated to project modulated sinusoidal fringes for detailed depth information. The coordinate systems of the four cameras can be unified using the calibration chessboard. The 3D scanner provides a detailed point cloud mesh of the biological sample surface, where each point was associated with a coordinate and normal vector in 3D space. Upon completing the morphological measurements, the sample holding plate was translated 200 mm linearly to align with the origin of the mechanical scanning platform.

(2) MUSE microscope: The MUSE microscope features a high‐speed Complementary Metal‐Oxide‐Semiconductor camera, an objective lens, a tubing lens, and UV LEDs. MUSE images were compared using both a color camera (VCC‐25CXP1R, CIS Inc.) and a monochrome camera (VCC‐25CXP1M, CIS Inc.), each offering identical pixel resolution and frame rates. The monochrome camera was selected for its higher sensitivity to fluorescent light (Figure S4, Supporting Information). The interchangeable objective lenses (M Plan Apo, Mitutoyo Inc.) feature a long working distance to accommodate irregular sample surfaces. To simultaneously excite Hoechst 33342 and Rhodamine B,^[^ ^30^ ^]^ six 265‐nm LEDs (HSE265H‐M807X‐V20, Hasunopto Inc.) with heat sinks were positioned around the objective lens. These LEDs were activated only during image acquisition to prevent overheating. The LED holder (Figure S11, Supporting Information) facilitates the concentration of UV energy within a 6.2 mm diameter region on the tissue surface.

(3) Mechanical scanning platform: The mechanical scanning platform enables rapid and precise scanning of the MUSE microscope around the sample. It consists of three linear stages (X, Y, Z) and three rotation stages (R_x_, R_y_, R_z_) (Figure S12, Supporting Information). The origin point (*O*) of the scanning platform's coordinate system was defined at the center of the calibration chessboard (Figure S13, Supporting Information), with the rotational axes of the R_x_‐, R_y_‐, and R_z_‐stages converging at this point. The microscope was mounted on a light‐duty linear stage (Z), allowing for scanning perpendicular to a specific region on the fluctuating sample surface and extending DOF through image processing. The Z‐stage was connected to two rotation stages (R_y_, R_x_), which are assembled using an L‐shaped aluminum bracket. The R_x_‐stage was further installed on a heavy‐duty linear stage (Y), providing precise control over the microscope's rotation and translation. The remaining stages (X and R_z_) enable the movement and rotation of both the calibration chessboard and biological samples. The calibration chessboard, securely positioned on the R_z_‐stage beneath the biological sample, features precisely printed patterns.

(4) Calibration chessboard and origin alignment: The calibration chessboard (DF0002, DFvision Inc.) features a high‐contrast alternating pattern of white and black squares (Figure S13, Supporting Information), enabling precise calculation of the 3D scanner's extrinsic matrix. A small central dot (0.1 mm) marks the coordinate origin (*O*), and 12 adjacent dots assist in determining the microscope's intrinsic and extrinsic matrices. The precisely defined patterns on the chessboard were visible to both the 3D scanner and the microscope, ensuring accurate alignment of the coordinate systems between the 3D scanner and the mechanical scanning platform.

### Scan Trajectory Planning

The mechanical scanning trajectory was automatically generated after morphological measurement by dividing the 3D mesh into square grids, each slightly smaller than the MUSE microscope's FOV. For each grid, the averaged normal vector and elevational scanning range were computed. These parameters informed the mechanical scanning protocol, guiding the microscope to the geometric center of each grid while aligning it parallel to the averaged normal vector. Scanning was performed along the normal direction, covering the elevational range in steps that matched the objective lens's DOF. The distance between neighboring grid centers was set to be ≈20% smaller than the microscope's FOV length (*l*) to ensure optimal overlap for image registration. Specifically, the FOV length was determined using the formula: *l = du*  ·  *pixel_N_ / m*, where *m* represents the microscope's magnification; *du* is the pixel size (4.5 µm) of the camera sensor, and *pixel_N_* is the number of pixels (5120) along one dimension of the square sensor. The detailed methodology is provided below:

1. Identifying the starting point: The 3D surface mesh data was initially loaded and processed to smooth the surface and eliminate isolated vertices. The centroid *P_o_* of the surface mesh was then calculated and assigned as the starting point for the scanning process.
2. Dividing the mesh into grids: A tangent plane at point *P_o_* on the 3D mesh was constructed using the K‐D tree algorithm to locate adjacent grids (Figure S14, Supporting Information).^[^ ^50^ ^]^ The normal vector ***n*** of the tangent plane was computed using the least squares method. Four reference normal vectors (***n_x+_***, ***n_x‐_***, ***n_y+_***, ***n_y__‐_***) were defined along the coordinate axes. The normal vector ***n*** was then cross‐multiplied with each of these reference vectors to yield four base normal vectors (***n_1_***, ***n_2_***, ***n_3_***, ***n_4_***) of the tangent plane. Subsequently, each base normal vector was scaled by a factor of 0.8 × *l* (grid length) to compute the centroids of neighboring grids. If a computed centroid fell outside the surface mesh, the nearest point on the surface was assigned as the actual centroid. This recursive process continued until the boundary of the 3D mesh was reached, which served as the termination condition.
3. Calculating positions and orientations of the microscope: For each grid, the centroid *P_i_* and the averaged normal vector within the grid determine the microscope's position and orientation. The linear stages aligned the microscope's FOV center with *P_i_*, while the rotation stages adjusted its orientation. From a mechanical scanning perspective, the microscope's orientation can be described using Z‐X‐Y Euler angles, where the third column of the Euler angle matrix defines the orientation and was used to compute the rotation angles for the R_x_‐ and R_y_‐stages.^[^ ^51^ ^]^ Once positioned at a grid, the microscope scanned from 0.2 mm below the lowest point to 0.2 mm above the highest point in the mesh grid, accommodating potential measurement errors from the 3D scanner. The optimal scanning path between grids was determined by minimizing rotation angles. To minimize space occupation during scanning, the coordinate system was divided into four quadrants on the *x‐y* plane. By rotating the R_z_‐stage (i.e., biological sample), the microscope scanning can be confined within a single quadrant.

### System Calibration

Given the potential for errors introduced during manufacturing and assembly, the RAM system requires comprehensive calibration to achieve micrometer‐scale precision in scanning and imaging. The calibration process consists of three key steps: microscope calibration, rotation stage calibration, and linear stage calibration.

(1) Microscope calibration: The initial step involves aligning the microscope's starting position to ensure its imaging plane was parallel to the calibration chessboard. Another task was to determine the microscope's effective magnification, accounting for any discrepancies in the distance between the tube lens and the camera sensor. These calibration tasks can be addressed by calculating both the intrinsic and extrinsic matrices of the microscope. The intrinsic matrix *A* represents the internal parameters of the microscope, while the extrinsic matrix *B* captures its orientation and position relative to the scene. Specifically, the microscope can be modeled as an infinity‐corrected optical system:^[^ ^52^, ^53^ ^]^

(1) $$A=\left[\begin{array}{ccc} \frac{m}{du} & 0 & 0\\ 0 & \frac{m}{du} & 0\\ 0 & 0 & 1 \end{array}\right]$$

(2) $$B=\left[\begin{array}{cccc} r_{11} & r_{12} & r_{13} & t_{x}\\ r_{21} & r_{22} & r_{23} & t_{y}\\ 0 & 0 & 0 & 1 \end{array}\right]$$

(3) H=A·B

Here, the homography matrix *H* is employed to map the pixels of microscopic images to the spatial coordinates on the mechanical scanning platform, which is defined by the calibration chessboard. Specifically, elements *r_11_* through *r_23_* in the extrinsic matrix define the microscope's orientation angles relative to the calibration chessboard, while *t_x_* and *t_y_* represent its spatial positions.^[^ ^53^ ^]^

Initially, the microscope was positioned roughly parallel to the calibration chessboard, with the assistance of a level gauge. The RAM system then imaged the 13 small dots on the chessboard (Figure S15A, Supporting Information) and calculated the effective magnification *m* using the bundle adjustment algorithm.^[^ ^54^ ^]^ Once *m* was determined, the microscope's intrinsic matrix *A* could be fully defined. Subsequently, the extrinsic matrix *B* was calculated iteratively by fine‐tuning the microscope's orientation and position until parallelism between the camera sensor and the calibration chessboard was achieved.

(2) Rotation stage calibration: This step addresses orientation mismatches between the intended and actual rotation axes of the R_x_‐, R_y_‐, and R_z_‐stages. Such misalignments can arise due to manufacturing tolerances, assembly errors, or mechanical deformations, which may displace the microscope's FOV centroid after rotation. The process started by aligning the central point *O* on the calibration chessboard with the FOV centroid. The R_x_‐ and R_y_‐stages were then incrementally rotated by a fixed angle, and at each step, the displacement between the FOV's centroid *O’* and the chessboard's center *O* was recorded (Figure S15B, Supporting Information). The pixel coordinates (*O_x_*, *O_y_*) of point *O* in each microscopic image were marked to calculate the pixel distance to the FOV centroid *O’*, located at (*pixel_N_ /2*, *pixel_N_ /2*). Using the rotation step angle, the estimated extrinsic matrix $\hat{B}$ was calculated based on the Euler angle matrix.^[^ ^51^ ^]^ This estimated extrinsic matrix, along with the intrinsic matrix *A*, allowed for the computation of the estimated homography matrix $\hat{H}$ for each rotation step. Consequently, the microscope's positional correction (*offset_x_*, *offset_y_, 1*) on the horizontal plane was calculated based on the centroid deviation:

(4) $$\left[\begin{array}{c} offset_{x}\\ offset_{y}\\ 1 \end{array}\right]={\left(A\cdot \hat{B}\right)}^{-1}\cdot \left[\begin{array}{c} O_{x}-\frac{pixel_{N}}{2}\\ O_{y}-\frac{pixel_{N}}{2}\\ 1 \end{array}\right]={\hat{H}}^{-1}\cdot \left[\begin{array}{c} O_{x}-\frac{pixel_{N}}{2}\\ O_{y}-\frac{pixel_{N}}{2}\\ 1 \end{array}\right]$$

To refine the calibration, linear interpolation was applied to correct the microscope's position between each rotation step, ensuring smooth adjustments throughout the full range of rotation.

We then corrected errors in the rotation axis of the R_z_‐stage, as its actual rotation axis may not perfectly align with the chessboard's central point *O*. To address this, the microscope was initially positioned so that its FOV centroid *O’* coincided with *O*. As the R_z_‐stage rotated clockwise in fixed‐angle increments, images of the calibration chessboard were captured. The locations of point *O* in these microscopic images were recorded and fitted to a circle (Figure S15C, Supporting Information), where the circle's center represented the actual rotation axis of the R_z_‐stage, and the radius indicated the offset from the ideal alignment.

(3) Linear stage calibration: This step compensates for alignment errors between the coordinate axes and the X‐ and Y‐stages. The microscope was scanned along the *x*‐ and *y*‐axes, with the incremental distance precisely matching the length of each square pattern on the chessboard (4 mm). At each step, the coordinates of the square vertices within the FOV were recorded and fitted to a diagonal line. The angle between this line and the *x*‐ or *y*‐axis revealed any alignment error in the X‐ and Y‐stages (Figure S15D, Supporting Information). The overall accuracy of the RAM system was significantly enhanced after calibration (Figure S16, Supporting Information).

### Image Reconstruction and Processing

Following the mechanical scanning of the biological sample, the RAM system acquired multiple clusters of 2D microscopic images. For each image cluster, several frames containing a mix of blurry and sharp pixels were captured at different elevations within the grid. The number of frames in each cluster, ranging from 10 to 30, was determined by the elevational variation within the grid and the DOF of the objective lens. A Laplacian pyramid‐based image fusion algorithm was employed to combine the in‐focused pixels from different frames,^[^ ^55^ ^]^ resulting in a clear 2D image. Following the DOF extension, histogram equalization was applied to mitigate illumination variations caused by surface fluctuations within the grid.

In the image synthesis algorithm, designed to map grid images onto the entire surface, 3D mesh points were first connected using the Poisson surface reconstruction algorithm to form triangular facets.^[^ ^56^ ^]^ Sharp image pixels were then projected onto these facets using the estimated homography matrix $\hat{H}$, ensuring accurate spatial alignment between the 2D images and the 3D surface. To minimize geometric misalignments and lighting discrepancies, view selection was employed using a pairwise Markov random field and brightness adjustment via Poisson editing,^[^ ^57^ ^]^ producing seamless image features in the 3D representation.

For image post‐processing, a virtual H&E rendering algorithm was implemented to facilitate clinical interpretations of the RAM images. To develop the virtual rendering model, a cycle‐consistent generative adversarial network (CycleGAN) architecture was applied, which enabled unpaired translation from RAM to H&E images.^[^ ^32^, ^58^ ^]^ Beyond the standard loss functions in CycleGAN, a custom Color Moment Loss method was introduced to ensure color consistency between MUSE and H&E images. The Color Moment Loss function *L_color_* is expressed as *L_color_ (RAM, HE) = MSE (Mean_RAM_, Mean_HE_) + MSE (Std_RAM_, Std_HE_)*. In this formula, *Mean_RAM_* and *Mean_HE_* represent the mean color values of the RAM and H&E images, while *Std_RAM_* and *Std_HE_* correspond to the standard deviations of the color values. The mean square error function (*MSE*) was employed to quantify the differences between the color vectors.

### Image Feature Extraction and Statistical Analysis

For pathological labeling, an exact one‐to‐one correspondence of individual subimages between RAM and histopathology slides was not required, as tissue deformation can occur during sectioning. Instead, histopathologists first identified and annotated positive and negative regions on the corresponding FFPE H&E sections. These annotated regions were then aligned with the RAM images along the vertical sectioning lines, with the start and end points of each region determined by the histopathologist (Figure S17, Supporting Information). To develop a CAD‐based model for cancer margin assessment, the aligned regions in RAM were subsequently segmented into 128 × 128‑pixel subimages (≈0.12 × 0.12 mm^2^), each inheriting the pathological label of its corresponding annotated region.

Four imaging features were extracted from each subimage for pathological analysis (Table S2, Supporting Information): nucleocytoplasmic ratio, average nuclear signal intensity, mean nuclear cross‐section area, and standard deviation of nuclear cross‐section area. To quantify these image features, cell nuclei were segmented using a K‐means clustering algorithm.^[^ ^59^ ^]^ This algorithm converted the images into binary format, allowing for the computation of nuclei coordinates, counts, and cross‐section areas. Scratches and hemorrhagic necrosis in the images were excluded from further analysis to mitigate potential confounding effects.

Based on the quantified image features, statistical analysis was conducted to evaluate the accuracy of tumor margin assessment. Subimages were randomly split into training and testing datasets with an ≈6:4 population ratio. Logistic regression was then applied to build the tumor margin assessment model using the extracted RAM image features.^[^ ^60^ ^]^ ROC curves were plotted by varying the decision thresholds for each feature individually, as well as their combination (Figure 4B). To establish the “ground truth” for the assessment, pathologists performed H&E staining on FFPE sections from the region of interest. Only subimages located within 0.5 mm of the sectioned slice on the tissue surface were included in the statistical analysis, ensuring close correspondence with FFPE H&E results.

### Tissue Sample Preparation

All animal studies followed the protocol (ZJU20230210) approved by the Laboratory Animal Welfare and Ethics Committee of Zhejiang University. 8‐week‐old female nude mice (Balb/c, 10–15 g body weight) and a NASH model mouse obtained from Vital River Inc were used. In this feasibility study, 4T1 (mouse breast cancer) and B16 (mouse melanoma) cells were selected to create positive tumor margins. For the subcutaneous tumor model, mice were sacrificed by cervical dislocation once the tumor reached a diameter of 10 mm, followed by excision of the tumor along its periphery. In the metastasis model, where positive tumor margins were not visually discernible, mice were sacrificed two weeks after intraperitoneal injection, and their livers and spleens were removed for RAM imaging. Following a 10 s staining process and a subsequent 10 s rinse with phosphate‐buffered saline, fresh tissue samples were rapidly imaged using the RAM system. Formalin fixation can be conducted to preserve the samples for additional imaging tests days after excision, without affecting image quality (Figure S18, Supporting Information).

All human sample preparation, imaging, and analysis were conducted in accordance with the protocol (IR2024535) approved by the Human Research Ethics Committee of the Second Affiliated Hospital, Zhejiang University School of Medicine. To facilitate tissue collection and minimize disruption to the traditional clinical workflow, the RAM system was positioned in an isolated area adjacent to the surgery suite. Immediately following excision of the skin lesions, fresh tissue samples were promptly transferred to the RAM system for nondestructive imaging. Prior to scanning, the tissue surface was sequentially stained with Hoechst 33342 (0.5 mg mL^−1^) and Rhodamine B (0.5 mg mL^−1^) for 5 s each, followed by a 10 s rinse with phosphate buffered saline. Notably, the entire process—including sample preparation and imaging—was completed within minutes and did not interfere with subsequent histopathological processing or clinical diagnosis. To perform a comparison with the gold‐standard histopathology, which routinely sections tissue samples along their longitudinal axis for skin tumor diagnosis, the same axis was identified on the 3D surface image acquired by RAM. Subimages measuring 128 × 128 pixels and located within 0.5 mm of this axis on the tissue surface were then extracted for statistical analysis.

## Conflict of Interest

The authors declare no conflict of interest.

## Author Contributions

Z.S. and X.M. contributed equally to this work. L. L. and X. Y. M. conceived and designed the study. L. L., Z. S., P. F., and K. H. designed and constructed the proof‐of‐concept and functional prototypes. Z. S., X. W. M., X. L., and R. C. tested and optimized the prototypes. Z. S., X. W. M., and Y. Y. performed the experiments. Z. S. and X. W. M. analyzed the data. J. L., X. J., Y. Z., and Q. C. provided support of pathological examinations. P. F., J. F., Y. L., H. J. L., X. Y., and H. Z. helped with biological sample preparation. L. L. and P. F. supervised the study. All authors wrote the manuscript.

## Supporting information



Supporting Information

Supplemental Movie 1

Supplemental Movie 2

## Data Availability

The data that support the findings of this study are available from the corresponding author upon reasonable request.
