# Supplementary material for: Rapid Arbitrary‐Shape Microscopy of Unsectioned Tissues for Precise Intraoperative Tumor Margin Assessment
Source: Adv Sci (Weinh). 2025 Nov 20;13(8):e11919. doi: 10.1002/advs.202511919 (PMC12884736; doi:10.1002/advs.202511919)
Supplement: Supplementary file 1 — Supporting Information [file ADVS-13-e11919-s001.pdf]

Supplementary Materials for

Rapid Arbitrary-Shape Microscopy of Unsectioned Tissues for Precise Intraoperative Tumor Margin Assessment

Zhicheng Shao *et al.*

Corresponding authors. Emails: manxy@zju.edu.cn, linliokok@zju.edu.cn

This PDF file includes:

Figs. S1 to S18
Tables S1 to S3
Movies S1 to S2

Other Supplementary Materials for this manuscript include the following:

Movies S1 to S2

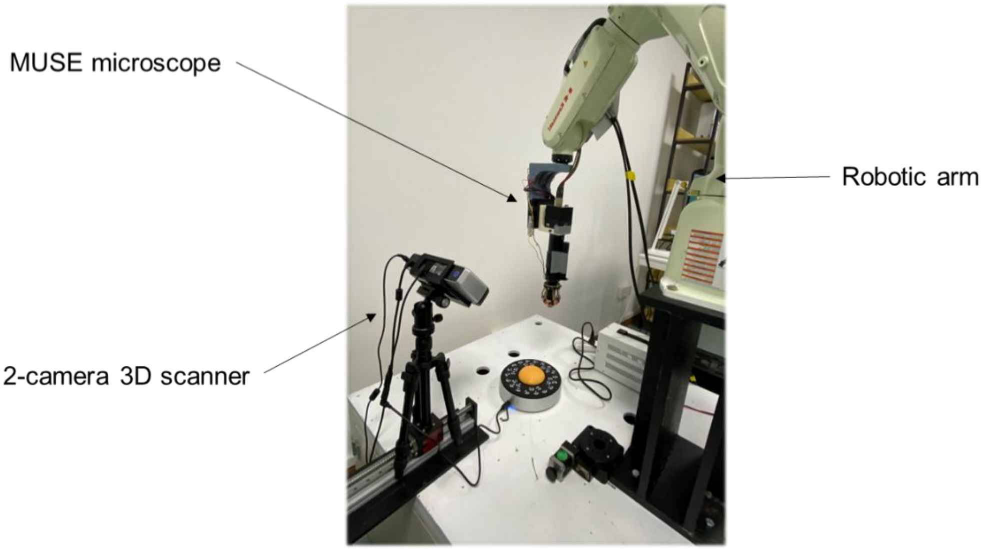

Figure S1. The proof-of-concept prototype. It primarily consists of a robotic arm (RS007N, Kawasaki Inc.), a 2-camera 3D scanner (EinScan-SP, Shining 3D Tech Co., Ltd.), and a customized MUSE microscope. In the functional prototype, both the robotic arm and the 3D scanner were replaced with customized modules to improve control and precision.

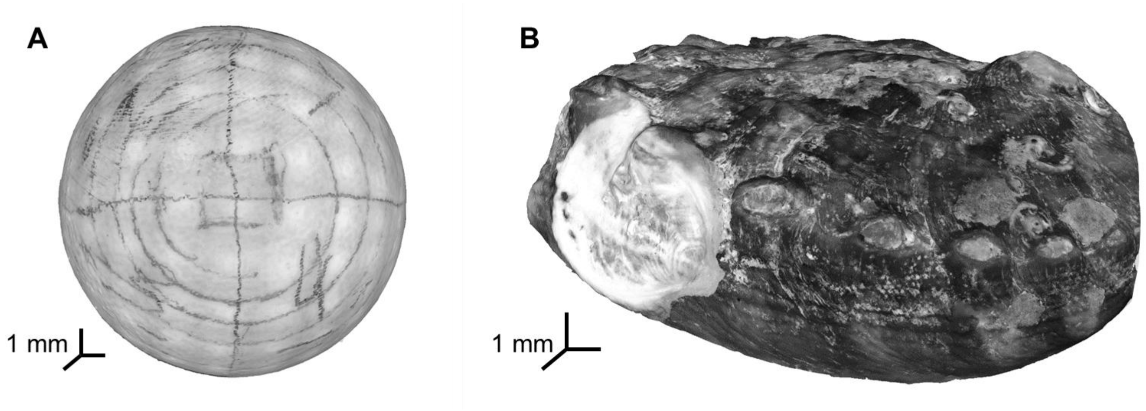

Figure S2. 3D phantom tests of the functional prototype performed prior to imaging biological tissues. (**A**) Top view of a 3D-printed hemisphere with surface patterns plotted. (**B**) 3D image of a seashell decoration. Both images were captured using a 5× objective lens under white light illumination.

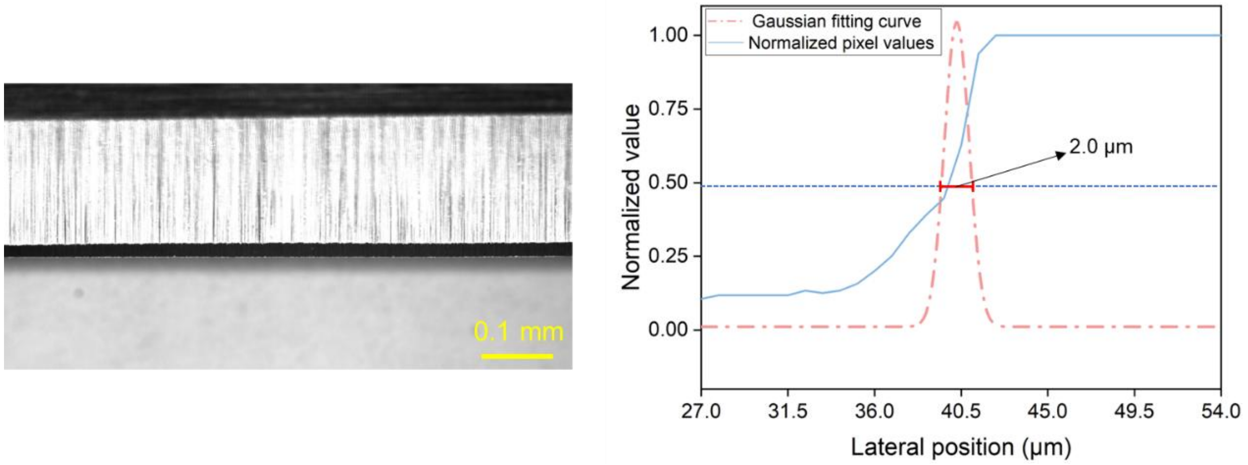

Figure S3. Spatial resolution measurement of the RAM system equipped with a 5× objective lens to image a blade. A lateral resolution of 2.0 micrometers was achieved.

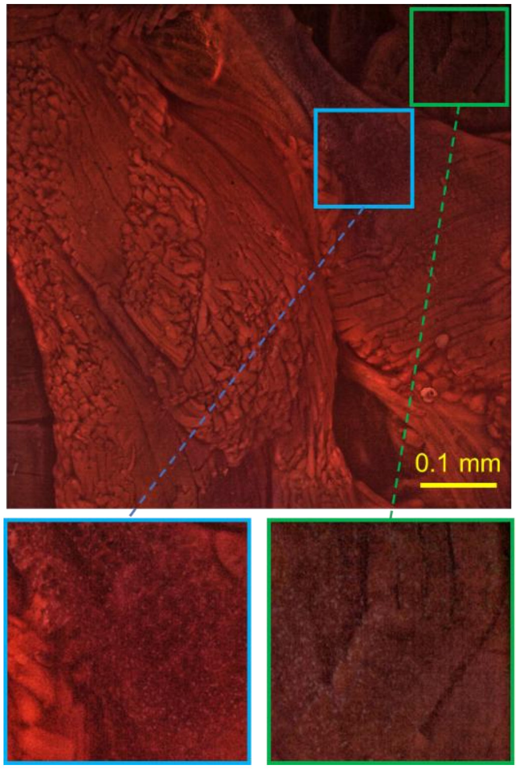

Figure S4. Side-by-side comparison of RAM images of swine tissue acquired with a color camera (left) and monochrome camera (right). The close-up images (bottom) highlight the superior signal-to-noise ratio of the monochrome camera, particularly in visualizing individual cell nuclei.

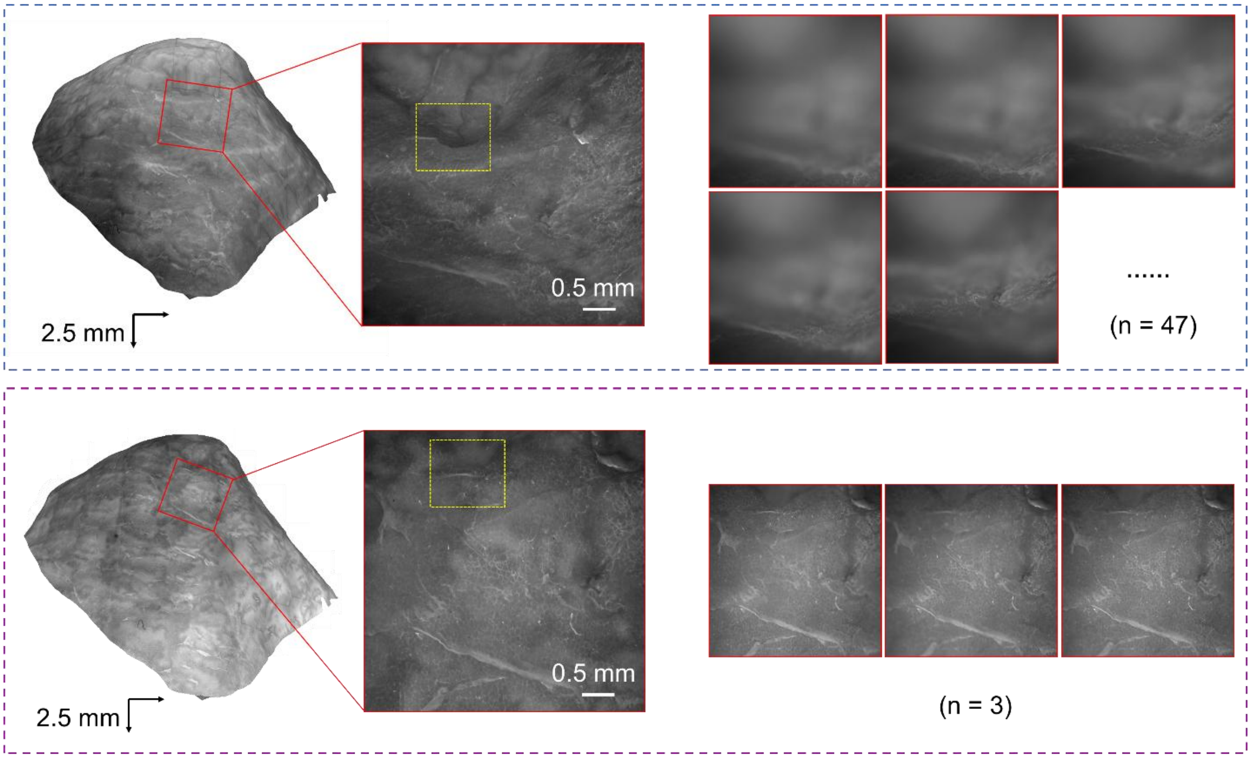

Figure S5. Comparison of images obtained by raster-scanned MUSE (top) and RAM (bottom) from a piece of swine tissue with an irregular surface. Equipped with a six-degree-of-freedom robotic platform, RAM can scan the unflattened surface along its local normal direction, reducing the number of scan steps and enhancing image clarity. In contrast, raster-scanned MUSE may fail to reveal features obscured by overlying tissue due to suboptimal viewing angles (regions highlighted by yellow dashed boxes).

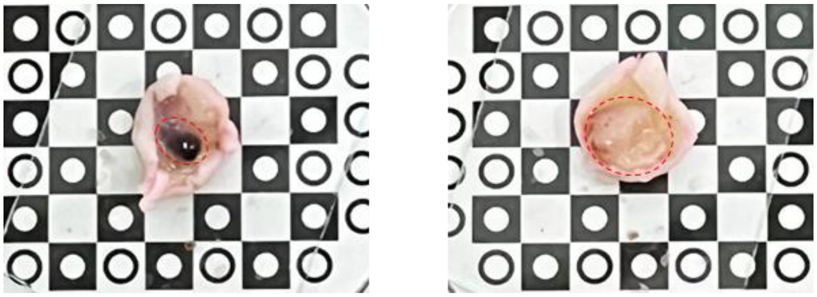

Figure S6. Photographs of subcutaneous melanoma (left) and breast tumor samples (right) on a glass slide positioned above the calibration chessboard. Visible tumor margins (highlighted in red dashed circles) are identifiable without the need for microscopic imaging.

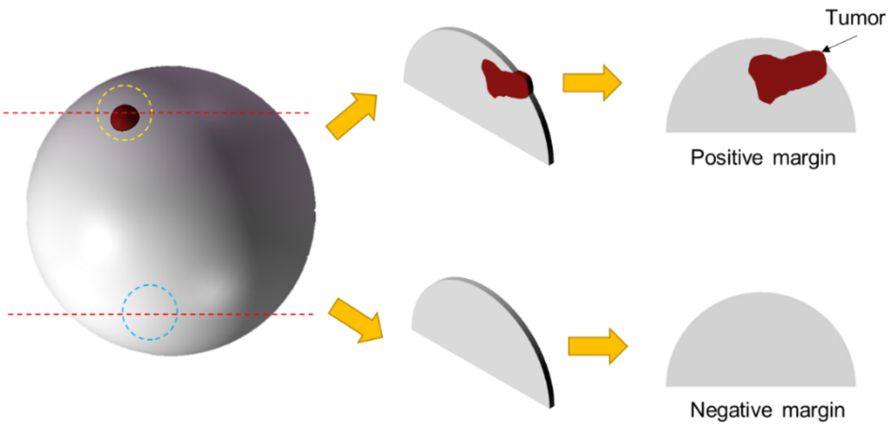

Figure S7. Illustration of the sectioning protocol for H&E comparison. All surface regions exhibiting suspicious RAM features (yellow dashed circle) are labeled, while regions without such features (blue dashed circle) are randomly labeled as negative areas. The tissue sample is then vertically sectioned across the labeled regions (red dashed lines) for FFPE H&E processing. Tissue slice boundaries are reviewed by independent pathologists to establish the "ground truth." The accuracy analysis of RAM focuses on tissue surface regions within a 0.5-mm distance from the sectioned slices (i.e., red dashed lines).

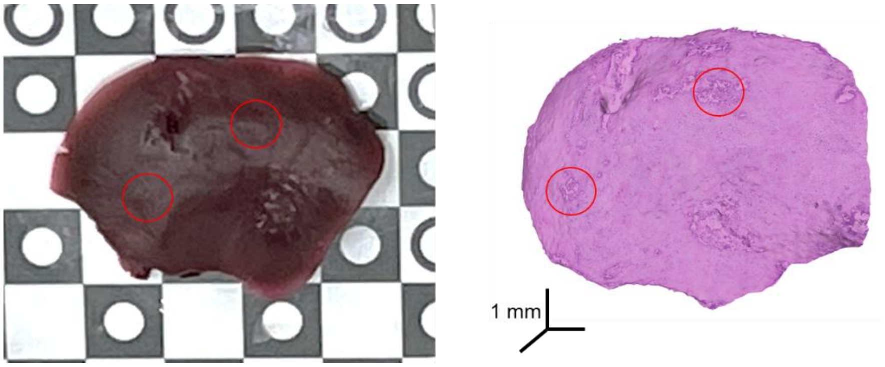

Figure S8. Photograph (left) and 3D RAM image (right) of a mouse liver sample with 4T1 cancer cell metastasis (highlighted in red circles). Although the metastatic tumor margins are not visible to the naked eye, they are clearly identifiable in the RAM image due to the higher nucleocytoplasmic ratios. Close-up views of the positive tumor margins and their confirmation with FFPE H&E staining are shown in Figure 3d–f (right column).

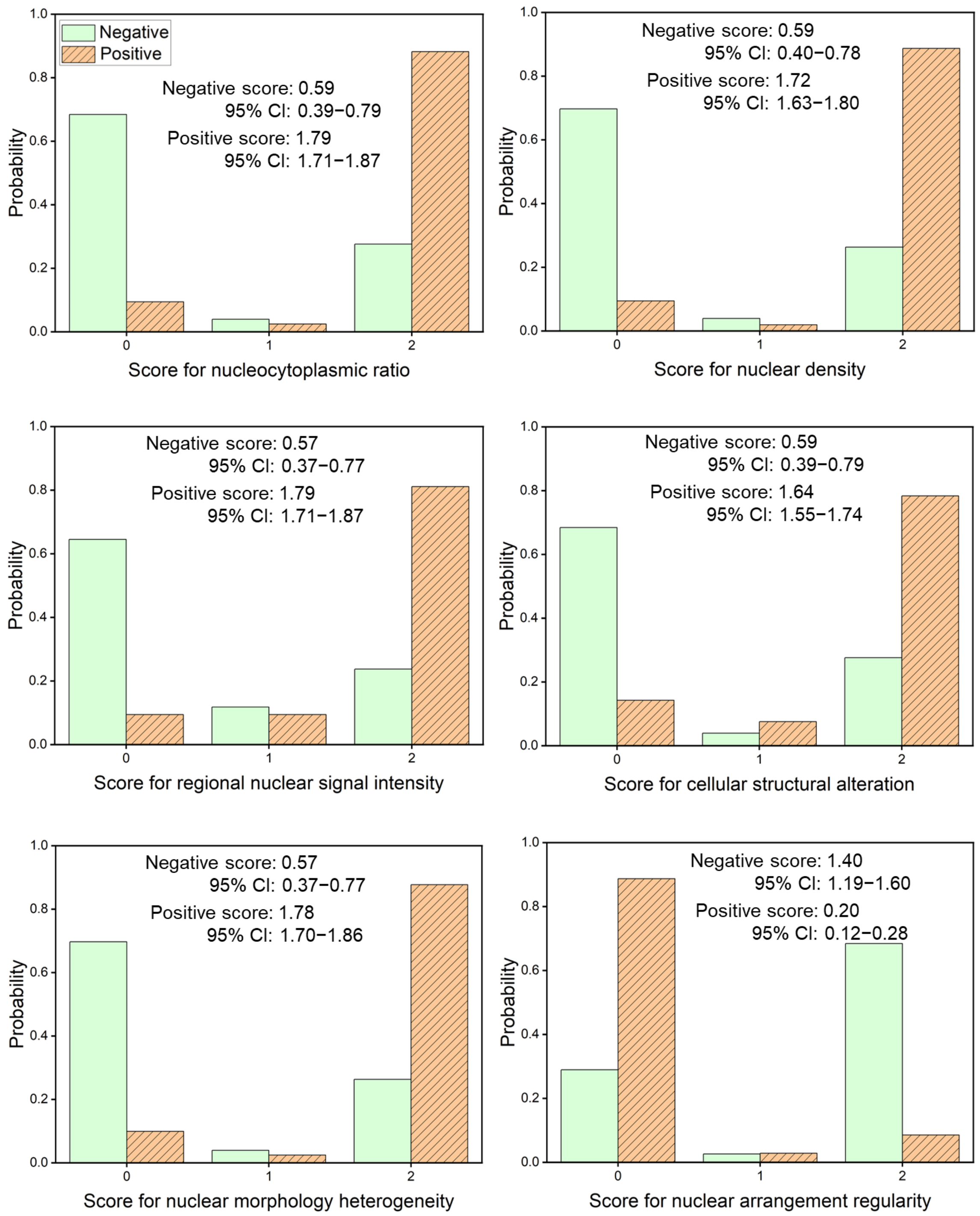

Figure S9. Histograms and mean score values of the image features graded by surgeon readers. Generally, a score of 0 indicates a low degree of feature presence, while a score of 2 represents a high degree. For example, in terms of cellular structural alteration, a score of 0 denotes inapparent alteration, whereas a score of 2 denotes apparent alteration. For nuclear arrangement regularity, a score of 0 represents an irregular arrangement, while a score of 2 signifies a regular arrangement.

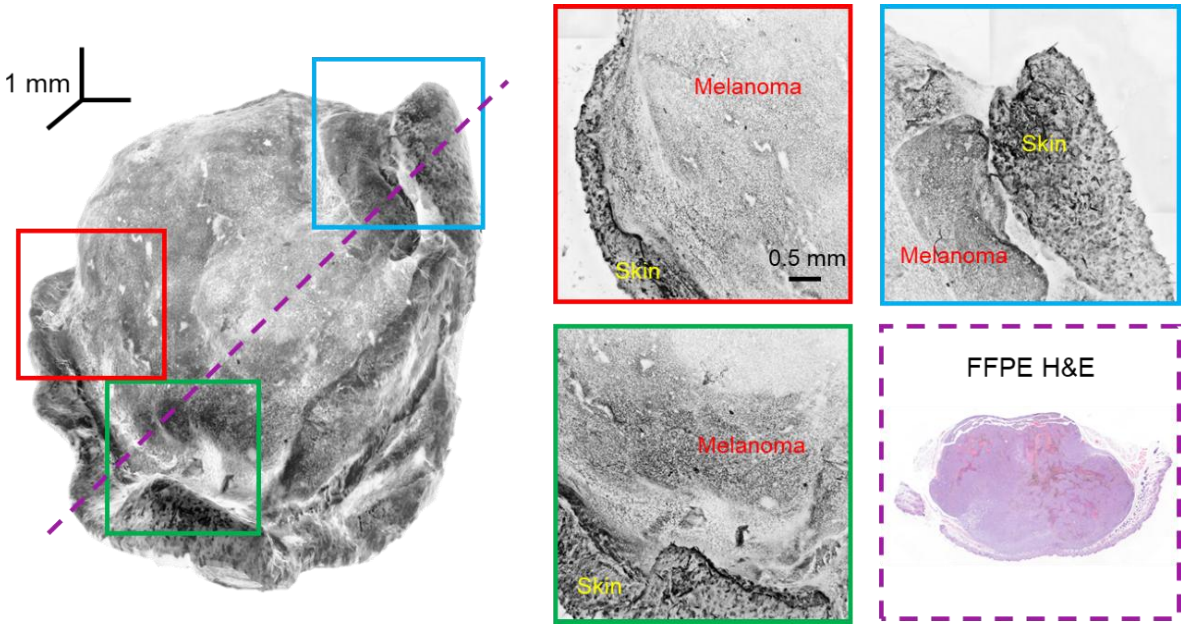

Figure S10. RAM images of a subcutaneous melanoma. The close-up images reveal differences in pathological structures between normal skin and melanoma cells. Tissue sectioning was performed along the dashed purple line for comparison with FFPE H&E staining. Please note that the close-up images may be viewed from different angles compared to the 3D full-surface image.

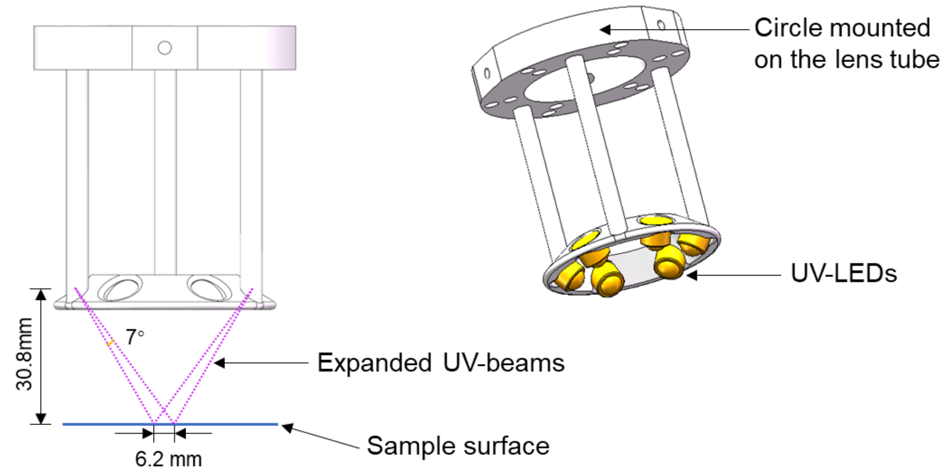

Figure S11. Schematic of the LED holder, which positions six UV-LEDs obliquely around the objective lens. Each LED has a divergence angle of 7 degrees, illuminating an area with a diameter of 6.2 mm on the sample surface, located 30.8 mm from the objective lens.

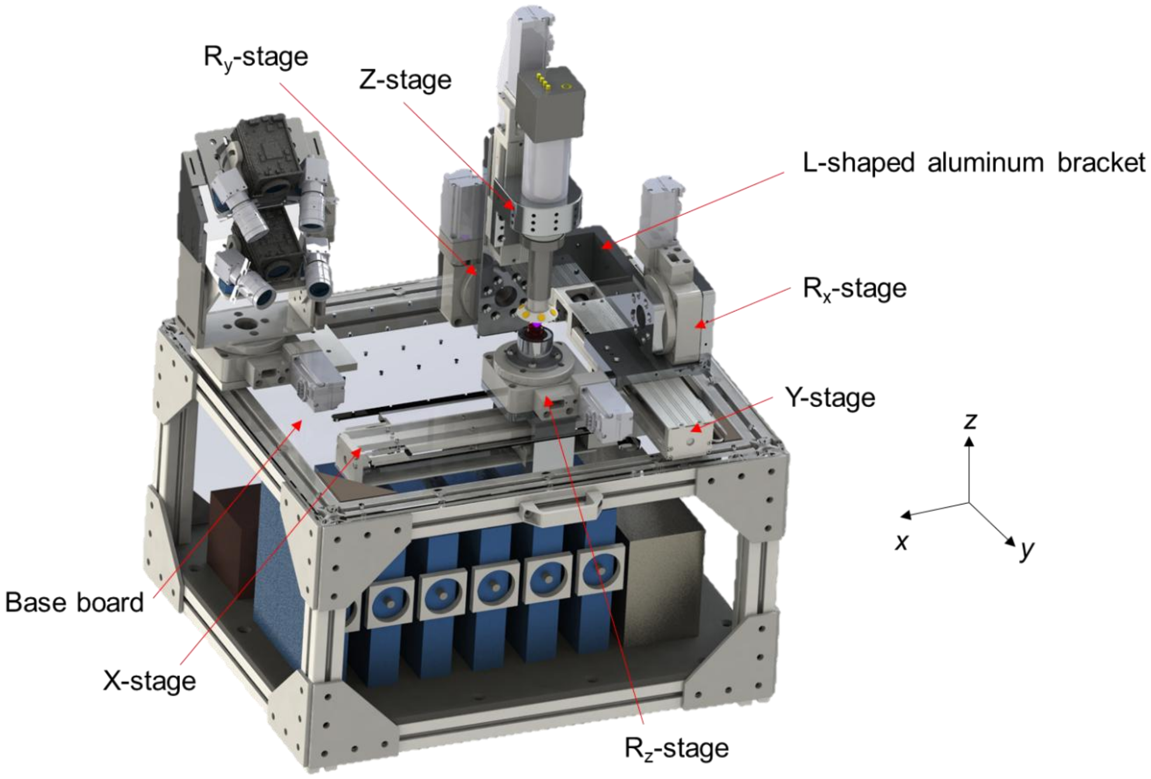

Figure S12. Perspective view of the functional prototype. The base board is rendered transparent to visualize the X-stage mounted underneath. The R_z-stage is mounted on the X-stage, allowing for both rotation and translation of the calibration chessboard and biological samples.

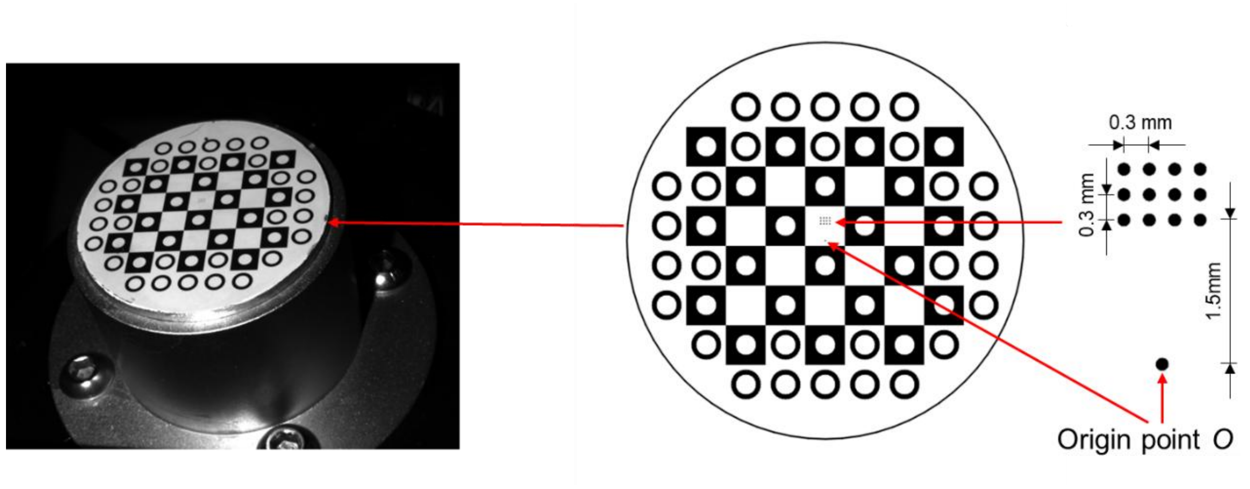

Figure S13. Photograph (left) and illustrations (right) of the calibration chessboard. Each black and white square on the chessboard measures 4×4 mm^2. A total of 13 black dots, each with a diameter of 0.1 mm, are printed in the central white square for system calibration. Specifically, the origin point O is marked at the geometric center of the calibration chessboard.

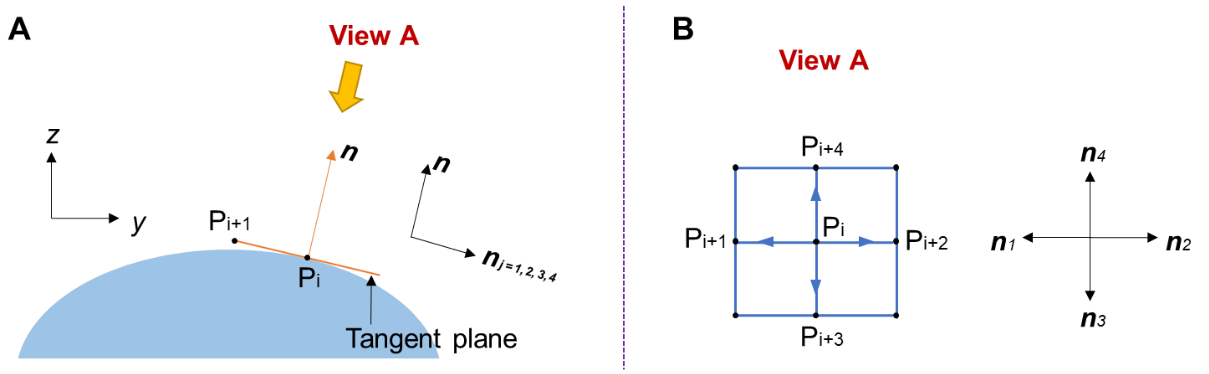

Figure S14. Illustration of the process for dividing a 3D surface mesh into square grids. (**A**) Cut-away view on the z-y plane showing a grid centroid P_i on the sample surface. The tangent plane at point P_i on the surface mesh is constructed using the K-D tree algorithm. (**B**) View against the normal direction $\boldsymbol{n}$ (View A). The centroids of neighboring grids are determined by calculating the base normal vectors of the tangent plane $\boldsymbol{n_1}$, $\boldsymbol{n_2}$, $\boldsymbol{n_3}$, and $\boldsymbol{n_4}$.

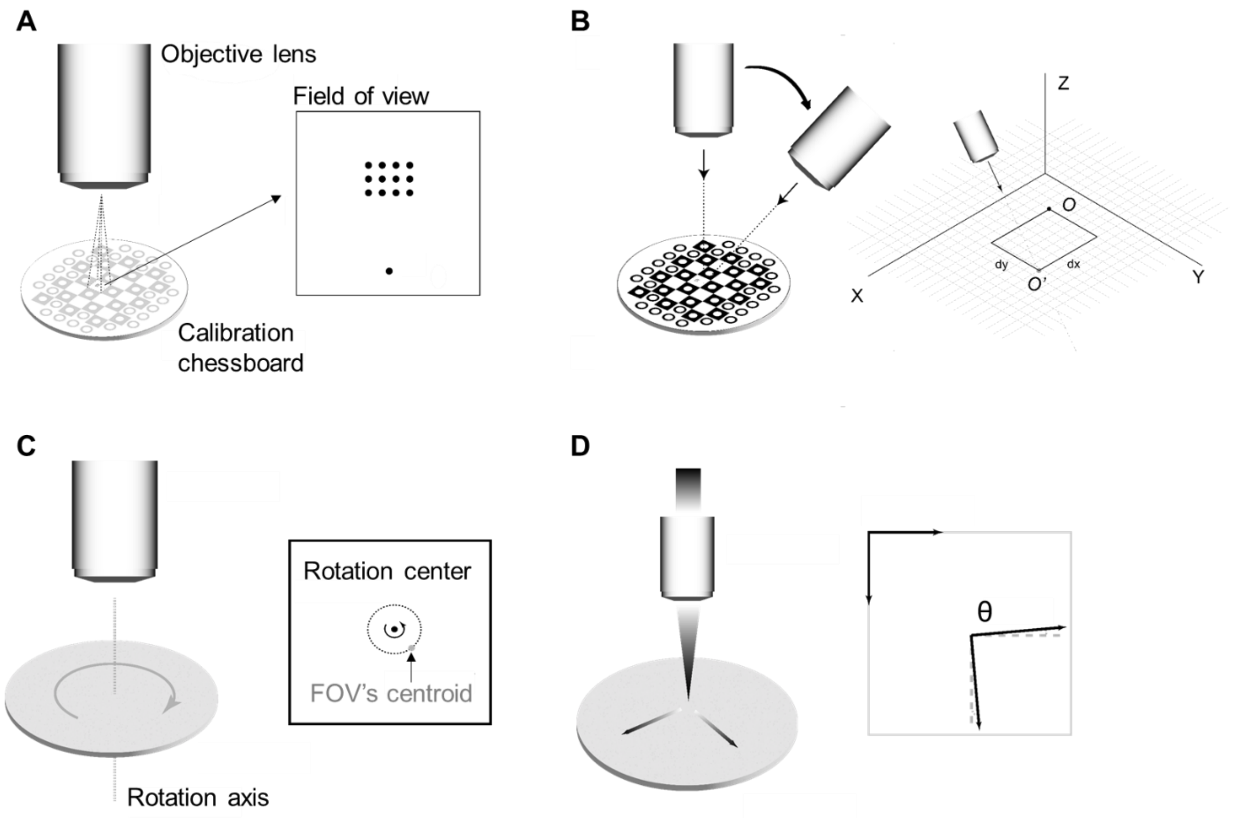

Figure S15. Illustration of the system calibration procedure. (**A**) Microscope calibration involves solving the extrinsic matrix and adjusting the microscope to ensure it is parallel to the calibration chessboard. (**B**) Calibration of the R_x- and R_y-stages is conducted by recording the distances between the centroids of the microscope's FOVs and the chessboard during rotation. (**C**) R_z-stage calibration is performed by measuring the misalignment between the rotation center and the centroids of microscope's FOVs. (**D**) Linear stage calibration is accomplished by measuring the angles between the scanning directions and the *x*- and *y*-axes defined by the calibration chessboard.

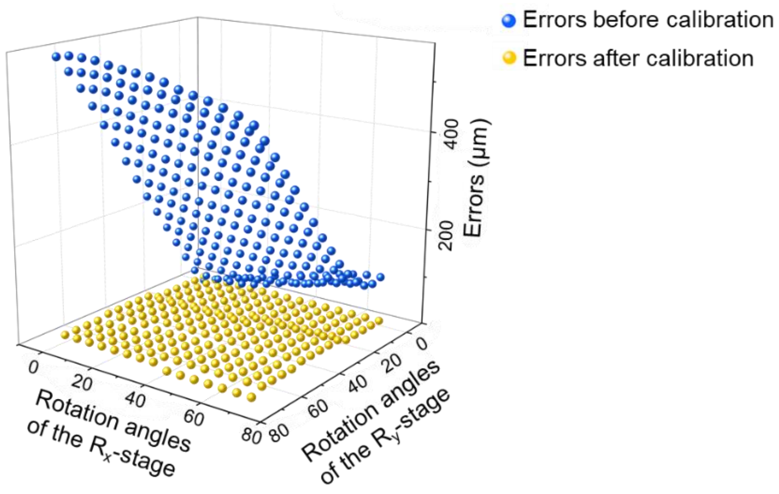

Figure S16. Geometric errors measured during the rotation of the R_x- and R_y-stages. They illustrate the mismatch between the origin point *O* and the microscope FOV's centroid *O'* before and after system calibration. The calibration significantly improves scanning accuracy.

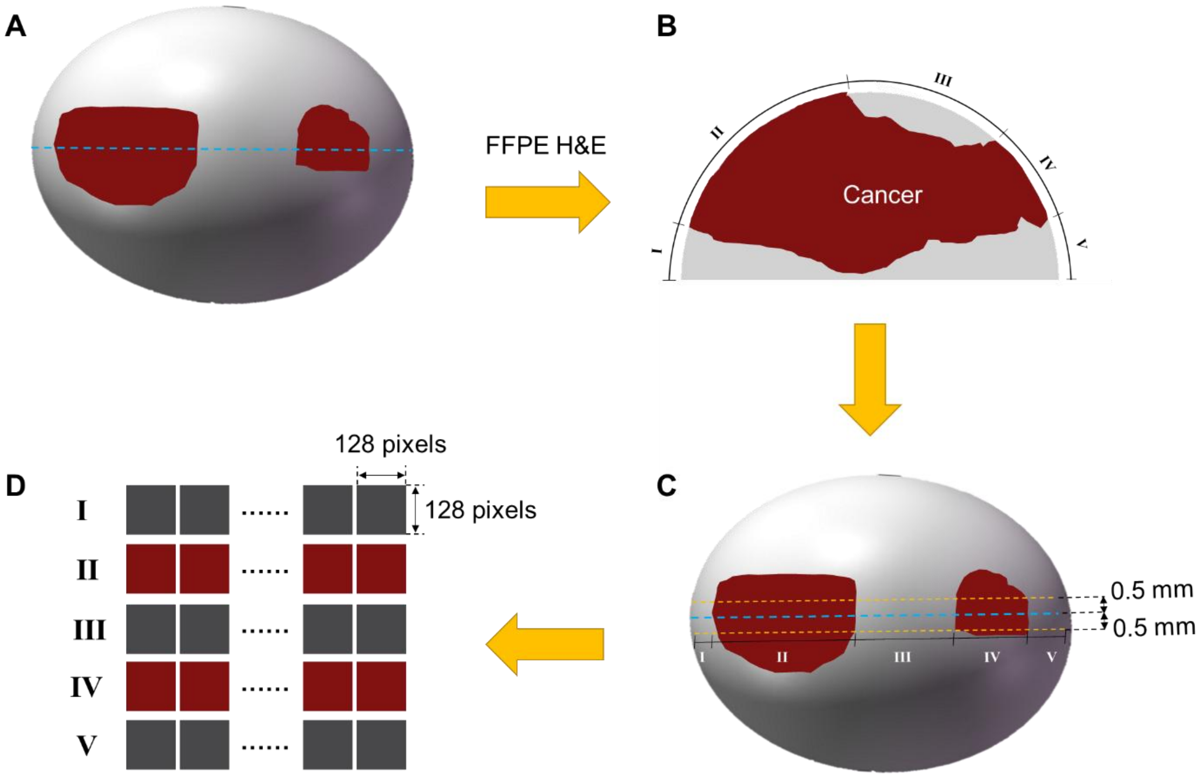

Figure S17. Workflow for pathological region annotation and alignment with RAM images. (A) Tissue specimen with positive tumor margins (red). The major axis is indicated by a blue dashed line. (B) The specimen was sectioned along its major axis for H&E staining and analysis. Histopathologists identified positive regions (e.g., Regions II and IV) and negative regions (e.g., Regions I, III, and V) along the sample margin. (C) In RAM images along the sectioning lines, histopathologists annotated each region based on alignment with the corresponding H&E slices. Yellow dashed lines indicate the subdividing area within 0.5 mm of the major axis. (D) Subimages derived from each annotated region inherit the pathological labels consistent with the H&E ground truth.

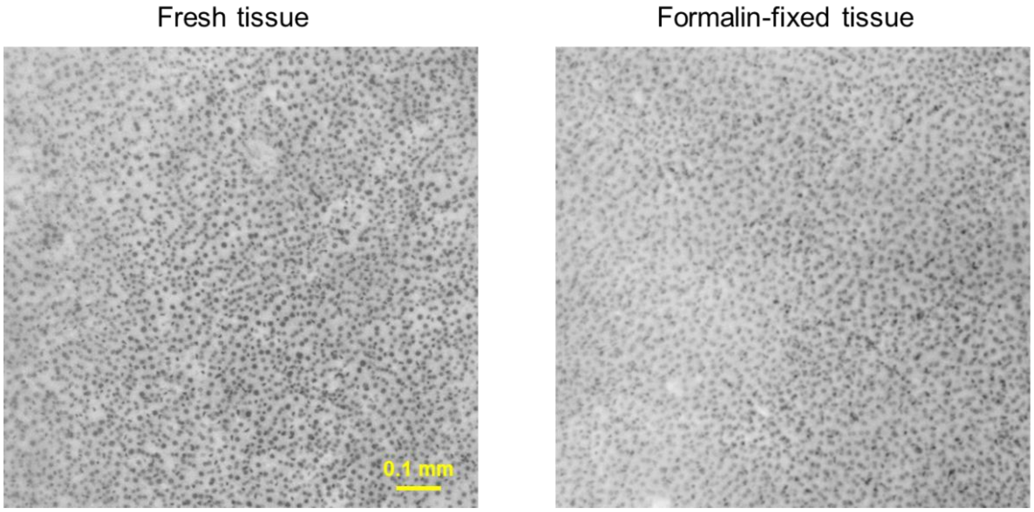

Figure S18. Comparison of RAM images acquired from different samples. A fresh liver sample (left) and the same liver sample after formalin fixation (right) demonstrate negligible differences in image quality before and after fixation.

Table S1. Description of the six RAM image features scored by surgeon readers. Since H&E-stained cell nuclei appear darker than the cytoplasm, we inverted the fluorescent signal intensities in the example images to facilitate interpretation.

Image feature	Description	Example image
Nucleocytoplasmic ratio	Because tumor cells typically have enlarged nuclei, they exhibit an increased nucleocytoplasmic ratio	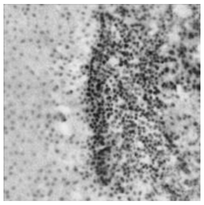
Regional nuclear signal intensity	The increased nucleocytoplasmic ratio and nuclear aggregation typically lead to enhanced fluorescent signal intensity at positive tumor margins	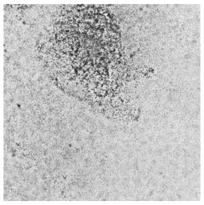
Nuclear density	Rapid tumor cell proliferation usually results in an increased nuclear density	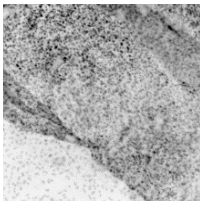
Cellular structural alteration	From a mesoscopic perspective, rapid tumor cell proliferation typically leads to a lump-like structure at positive tumor margins	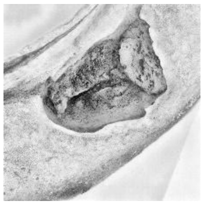
Nuclear morphology heterogeneity	Changes in tumor cell nuclear structures are reflected in an increased heterogeneity of nuclear morphology	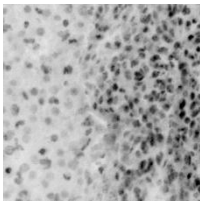
Nuclear arrangement regularity	The disorderly proliferation of tumor cells results in an irregular arrangement of nuclei	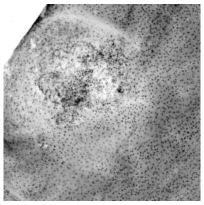

Table S2. Calculation equations for the four image features utilized in the CAD-based approach for tumor margin assessment.

Image feature	Calculation equation
Nucleocytoplasmic ratio	$\frac{\text{Number of pixels in the nuclei}}{\text{Number of pixels in the subimage} - \text{Number of pixels in the nuclei}}$
Average nuclear signal intensity	$\frac{\sum \text{Pixel intensity in the nuclei}}{\text{Number of pixels in the nuclei}}$
Mean nuclear cross-section area	$\frac{\text{Number of pixels in the nuclear region}}{\text{Number of nuclei in the subimage}}$
Standard deviation of nuclear cross-section area	$\left(\sum_{i=1}^{n} (i \text{ th nuclear cross-section area} - \text{Mean nuclear cross-section area})^2 \right)^{0.5}$

Table S3. Summary of key imaging and sample metrics from the clinical study, including tumor types, sample dimensions, RAM scanning time, and the ratio of cancer-positive to negative margin areas along the surgical surface's long axis as measured in RAM images. The efficiency of RAM scanning was influenced not only by sample size but also by the surface flatness.

Sample ID	Patient ID	Tumor type	Major axis length (mm)	Minor axis length (mm)	Microscope scanning time (min)	Positive / negative margin area ratio
1	1	Spindle cell tumor	20.27	13.63	6.9	2.07
2	2	Basal cell carcinoma	7.59	4.18	4.0	1.14
3	3	Keratoacanthoma	11.11	6.48	4.3	0.25
4	4	Epithelial carcinoma	9.85	6.59	2.4	0.45
5	5	Squamous cell carcinoma	8.47	7.09	2.2	1.43
6	6	Malignant melanoma	12.64	6.91	2.6	0.1
7	7	Basal cell carcinoma	13.29	8.72	2.9	1.49
8	8	Squamous cell carcinoma	13.55	12.3	3.1	3.58
9	9	Basal cell carcinoma	17.16	10.87	3.2	0.57
10	10	Malignant mesenchymal tumor	20.08	9.97	4.6	5.35
11	10	Malignant mesenchymal tumor	18.77	11.84	5.1	No negative margins
12	10	Malignant mesenchymal tumor	15.23	8.02	3.5	No negative margins

Movie S1. Workflow of RAM scanning, illustrating each operational step along with corresponding duration. The video is displayed at 8× speed for accelerated presentation.

Movie S2. A 3D rendered image of a mouse liver sample with positive tumor margins. Following the identification of high-risk regions through rapid gross scanning, the microscope can be switched to a higher magnification for detailed close-up observation.
